# LEGEND: Identifying Co-expressed Genes in Multimodal Transcriptomic Sequencing Data

**DOI:** 10.1093/gpbjnl/qzaf056

**Published:** 2025-07-01

**Authors:** Tao Deng, Mengqian Huang, Kaichen Xu, Yan Lu, Yucheng Xu, Siyu Chen, Nina Xie, Qiuyue Tao, Hao Wu, Xiaobo Sun

**Affiliations:** School of Data Science, The Chinese University of Hong Kong, Shenzhen (CUHK-Shenzhen), Shenzhen 518172, China; Shenzhen Research Institute of Big Data, Shenzhen 518172, China; School of Statistics and Mathematics, Zhongnan University of Economics and Law, Wuhan 430073, China; School of Statistics and Mathematics, Zhongnan University of Economics and Law, Wuhan 430073, China; School of Statistics and Mathematics, Zhongnan University of Economics and Law, Wuhan 430073, China; School of Statistics and Data Science, Nankai University, Tianjin 300071, China; School of Statistics and Mathematics, Zhongnan University of Economics and Law, Wuhan 430073, China; Department of Geriatric Neurology, Xiangya Hospital, Central South University, Changsha 410008, China; National Clinical Research Center for Geriatric Disorders, Changsha 410078, China; School of Statistics and Mathematics, Zhongnan University of Economics and Law, Wuhan 430073, China; Faculty of Computer Science and Control Engineering, Shenzhen University of Advanced Technology, Shenzhen 518055, China; Shenzhen Institute of Advanced Technology, Chinese Academy of Sciences, Shenzhen 518055, China; Department of Human Genetics, Emory University, Atlanta, GA 30322, USA

**Keywords:** Spatially resolved transcriptomics, Single-cell RNA sequencing, Co-expressed gene clustering, Gene co-functionality, Feature gene selection

## Abstract

Identifying co-expressed genes across tissue domains and cell types is essential for revealing co-functional genes involved in biological or pathological processes. While both single-cell RNA sequencing (scRNA-seq) and spatially resolved transcriptomics (SRT) data offer insights into gene co-expression patterns, current methods typically utilize either data type alone, potentially diluting the co-functionality signals within co-expressed gene groups. To bridge this gap, we introduce muLtimodal co-Expressed GENes finDer (LEGEND), a novel computational method that integrates scRNA-seq and SRT data for identifying groups of co-expressed genes at both cell type and tissue domain levels. LEGEND employs an innovative hierarchical clustering algorithm designed to maximize intra-cluster redundancy and inter-cluster complementarity, effectively capturing more nuanced patterns of gene co-expression and spatial coherence. Enrichment and co-function analyses further showcase the biological relevance of these gene clusters and their utilities in exploring context-specific novel gene functions. Notably, LEGEND can reveal shifts in gene–gene interactions under different conditions, providing insights into disease-associated gene crosstalk. Moreover, LEGEND can enhance the annotation accuracy of both spatial spots in SRT and single cells in scRNA-seq, and serve as a pioneering tool for identifying genes with designated spatial expression patterns. LEGEND is available at https://github.com/ToryDeng/LEGEND.

## Graphical abstract

**Figure:**
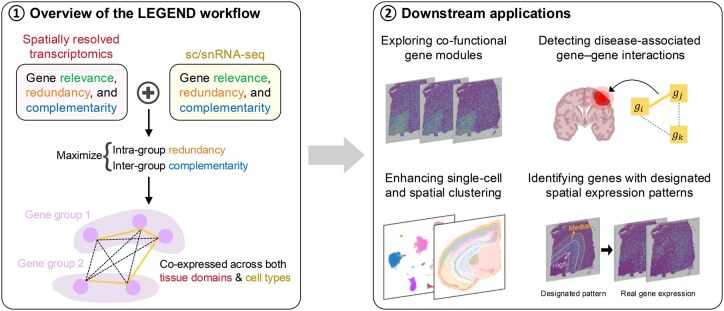

## Introduction

Single-cell RNA sequencing (scRNA-seq) technology allows the profiling of the whole transcriptome at individual cell resolution, yielding rich information on transcriptional activities among diverse cell types and gene expression dynamics in complex tissues and diseases [1–3]. However, the scRNA-seq data lack spatial information on single cells, which hinders the understanding of gene functions in the context of tissue microenvironment. Spatially resolved transcriptomics (SRT) addresses this issue by profiling spatial gene expression in tissues, thus providing unprecedented opportunities to characterize spatial distribution of cell types [4], delineate spatial tissue organization [5], explore gene–gene interactions [6], *etc*. Nonetheless, current high-throughput SRT technologies typically have to trade single-cell resolution for full transcriptome coverage (*e.g.*, *in situ* capturing-based technologies such as 10X Visium [7]), making it difficult to reliably determine single-cell identities at each measurement site (*i.e.*, spatial spot). Therefore, the integrative analysis of SRT and scRNA-seq data that utilizes their complementary information can significantly facilitate analytical tasks such as identifying gene expression patterns at both tissue domain and cell type levels.

The identification of gene expression patterns is essential for revealing gene functions and interactions involved in biological processes (BPs), including cell differentiation and tissue development. In the context of scRNA-seq and SRT, researchers focus on three categories of gene expression patterns: differentially expressed genes (DEGs), variably expressed genes [including highly variable genes (HVGs) in scRNA-seq and spatially variable genes (SVGs) in SRT], and co-expressed genes [5,8]. They provide different insights into the underlying BPs and genetic mechanisms. For instance, DEGs facilitate the discovery of cell type-specific/tissue region-specific biomarkers; variably expressed genes allow the detection of genes involved in the differentiation of cell types and tissue domains [8,9]; co-expressed genes can reveal functionally related genes and pathways [10,11], providing genetic clues to disease pathogenetic mechanisms based on their co-expression patterns associated with the development of pathology [12]. Intriguingly, it has been suggested that co-expressed genes can serve as genomic “contexts” for learning distributed gene representations to facilitate downstream analytical tasks, similar to the word context used in word2vec [13,14].

A variety of computational methods have been developed for identifying gene expression patterns in scRNA-seq and SRT [9,15–18]. Specifically, methods for identifying co-expressed genes include scGeneClust [11], cell-type-specific co-expressions (CS-CORE) [19], and CO-expression Tables ANalysis (COTAN) [20] for scRNA-seq, and convolutional neural network with protein–protein interaction-graph regularization (CNN-PReg) [10], Giotto [15], STUtility [21], and spatial pattern recognition via kernels (SPARK) [22] for SRT. However, these methods are constrained by several limitations. First, they work on SRT or scRNA-seq data alone and analyze gene co-expression solely across cell types or tissue domains. This leads to the identification of weaker co-functional genes in that they may exhibit similar expression patterns across tissue domains but not across cell types, or *vice versa*. Second, all methods for SRT except CNN-PReg evaluate gene expression similarity for each spot independently, neglecting the spatial relationships among spatial spots and the overall spatial patterns. Third, these methods fall short of offering means to leverage identified co-expressed genes for downstream applications, such as pinpointing genes with targeted spatial expression patterns or improving the informational efficiency of feature genes used by analytical algorithms.

**Figure 1:**
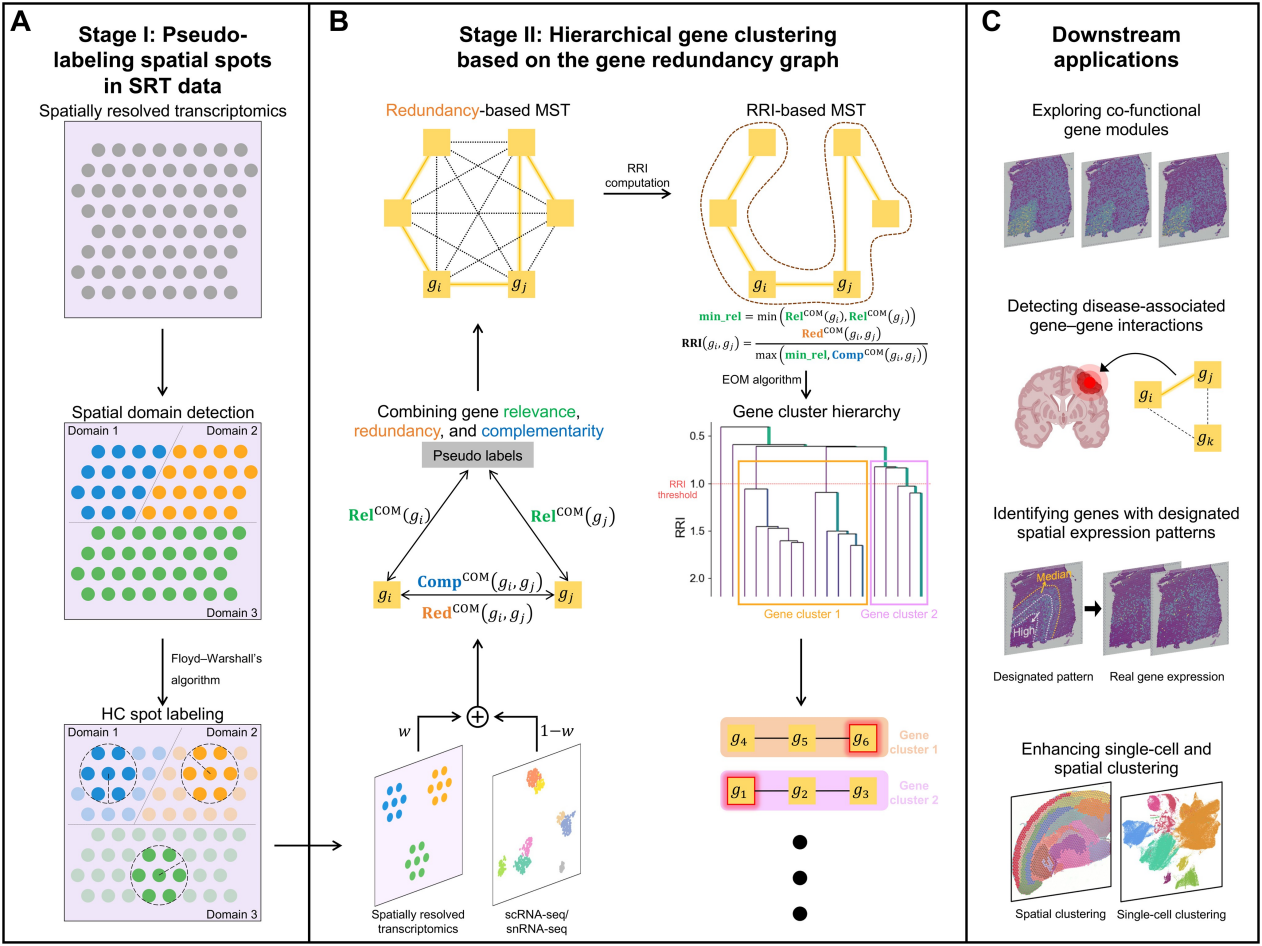
The workflow of LEGEND LEGEND consists of two stages. **A**. Pseudo-labeling spatial spots in SRT data. LEGEND initially groups spatial spots into spatial domains using an off-the-shelf spatial clustering algorithm. Floyd–Warshall’s algorithm is then employed to identify domain central areas wherein spots (*i.e.*, HC spots) reliably belong to the same domain. HC spots are used in subsequent steps, while domain marginal spots are excluded. **B**. Hierarchical gene clustering based on the gene redundancy graph. Gene relevance, redundancy, and complementarity are computed in both SRT and scRNA-seq datasets based on pseudo-labels of HC spots and HC cells (if true single-cell labels are unavailable). These metrics are then combined as weighted average composite values. A complete gene redundancy graph is then constructed, with edge weights equal to the composite redundancy between connecting gene pairs. From this graph, an MST is extracted and converted into a hierarchy of gene clusters through the EOM algorithm. Within this hierarchy, high-quality gene clusters, representing groups of co-expressed genes, are automatically determined based on their cluster stability coefficients. **C**. Gene clustering-based downstream applications. LEGEND-mediated gene clustering allows exploring co-functional gene modules, detecting disease-associated gene–gene interactions, pinpointing genes with designated spatial expression patterns, and enhancing both single-cell and spatial clustering. SRT, spatially resolved transcriptomics; HC, highly confident; scRNA-seq, single-cell RNA sequencing; snRNA-seq, single-nucleus RNA sequencing; MST, maximum spanning tree; LEGEND, muLtimodal co-Expressed GENes finDer; RRI, relative redundancy index; EOM, Excess of Mass.

In response, we developed a novel method, muLtimodal co-Expressed GENes finDer (LEGEND), which identifies co-expressed genes across both cell types and tissue domains under the framework of information theory (**Figure 1**). LEGEND estimates gene relevance, redundancy, and complementarity in both SRT and scRNA-seq datasets in a pseudo-semi-supervised manner. This information is used to construct a gene–gene redundancy graph, upon which genes are grouped as per their redundancy into hierarchical clusters that represent co-expressed and co-functional gene modules. We evaluated LEGEND over SRT and scRNA-seq datasets from adult mouse brain and human dorsolateral prefrontal cortex (DLPFC) (**Table 1**), and found that genes within the same cluster exhibit similar spatial expression patterns across all SRT datasets. In comparison to seven existing methods (**Table 2**) and an adapted variant of LEGEND that omits scRNA-seq data usage (referred to as LEGEND-no-sc), LEGEND shows superior gene clustering performance in terms of gene co-expression across cell types and spatial coherence across tissue domains. Additionally, via pathway enrichment analysis and gene co-function analysis, we found that LEGEND effectively groups context-specific co-functional genes into clusters, with aggregated expression patterns overlapping with tissue anatomical architectures and pathological distributions. Furthermore, we demonstrated LEGEND’s application in three key downstream tasks: identifying disease-associated differential gene co-expression, pinpointing genes with designated spatial expression patterns, and improving the accuracy of both cell clustering in scRNA-seq and spatial clustering in SRT. Real data analyses showcase our method’s superior performance across all these tasks and promise its potential in revealing gene-level pathogenic mechanisms.

**Table 1:** Datasets used in this study

Data type	Dataset	Species	Health status	Tissue	Technology	No. of spots/cells	Source	Task
SRT	mBrain-SRT	Mouse	Healthy	Brain	10X Visium	2688	10X Genomics	a, e, h
SRT	mBrain-FFPE	Mouse	Healthy	Brain	10X Visium FFPE	2438	10X Genomics	a
SRT	mBrain-HD	Mouse	Healthy	Brain	10X Visium HD	98,917	10X Genomics, 16 µm resolution	a
SRT	hDLPFC-SRT (slice 151507)	Human	Healthy	DLPFC	10X Visium	4226	Maynard et al. [23], spatialLIBD	a, e, h
SRT	hDLPFC-SRT (slice 151508)	Human	Healthy	DLPFC	10X Visium	4384	Maynard et al. [23], spatialLIBD	a, e, h
SRT	hDLPFC-SRT (slice 151509)	Human	Healthy	DLPFC	10X Visium	4789	Maynard et al. [23], spatialLIBD	a, d, e, h
SRT	hDLPFC-SRT (slice 151510)	Human	Healthy	DLPFC	10X Visium	4634	Maynard et al. [23], spatialLIBD	a, e, h
SRT	hDLPFC-SRT (slice 151669)	Human	Healthy	DLPFC	10X Visium	3661	Maynard et al. [23], spatialLIBD	a, e, h
SRT	hDLPFC-SRT (slice 151670)	Human	Healthy	DLPFC	10X Visium	3498	Maynard et al. [23], spatialLIBD	a, e, h
SRT	hDLPFC-SRT (slice 151671)	Human	Healthy	DLPFC	10X Visium	4110	Maynard et al. [23], spatialLIBD	a, e, h
SRT	hDLPFC-SRT (slice 151672)	Human	Healthy	DLPFC	10X Visium	4015	Maynard et al. [23], spatialLIBD	a, e, h
SRT	hDLPFC-SRT (slice 151673)	Human	Healthy	DLPFC	10X Visium	3639	Maynard et al. [23], spatialLIBD	a, c, e, g, h
SRT	hDLPFC-SRT (slice 151674)	Human	Healthy	DLPFC	10X Visium	3673	Maynard et al. [23], spatialLIBD	a, e, h
SRT	hDLPFC-SRT (slice 151675)	Human	Healthy	DLPFC	10X Visium	3592	Maynard et al. [23], spatialLIBD	a, e, h
SRT	hDLPFC-SRT (slice 151676)	Human	Healthy	DLPFC	10X Visium	3460	Maynard et al. [23], spatialLIBD	a, e, h
SRT	hMTG-health-SRT (sample 1-1)	Human	Healthy	MTG	10X Visium	3742	Chen et al. [12], GSE220442	a, b, c
SRT	hMTG-AD-SRT (sample 2-3)	Human	AD	MTG	10X Visium	4348	Chen et al. [12], GSE220442	a, b, c
SRT	hMTG-health-SRT2 (sample 144105)	Human	Healthy	MTG	10X Visium	3529	Omidsalar et al. [24], GSE226663	c
SRT	hBC-SRT	Human	BC	Primary breast tumor tissue	10X Visium	3798	10X Genomics	a
scRNA-seq	mCortex-sc	Mouse	Healthy	Anterior lateral motor cortex and primary visual cortex	Smart-Seq	23,822	Tasic et al. [25], GSE115746	a, f, h
scRNA-seq	hCortex-sc	Human	Healthy	Multiple cortical areas	Smart-Seq and Chromium	49,495	Jones et al. [26], Allen Human Brain Atlas	a, f, h
scRNA-seq	hMTG-health-sc	Human	Healthy	MTG	Smart-Seq and Chromium	7786	Jones et al. [26], Allen Human Brain Atlas	a, b, c
scRNA-seq	hMTG-AD-sc	Human	AD	MTG	Chromium	21,128	Jones et al. [26], Allen Human Brain Atlas	a, b, c
scRNA-seq	hMTG-health-sc2 (sample HC14MTG)	Human	Healthy	MTG	Chromium	5474	Zhang et al. [27], GSE188545	c
scRNA-seq	hPC-health-sc (sample NC3)	Human	Healthy	Prefrontal cortex	Chromium	6327	Lau et al. [28], GSE157827	c
scRNA-seq	hBC-sc (sample CID4471)	Human	BC	Primary breast tumor tissue	Chromium	8609	Wu et al. [29], GSE176078	a

*Note*: a, identifying co-expressed genes; b, biological analyses; c, gene–gene interaction prediction; d, detecting genes with designated spatial expression patterns; e, improving spatial clustering; f, improving single-cell clustering; g, identifying highly confident spots; h, impact of the number of selected feature genes on cell/spot clustering. In dataset names, the prefix “m” represents “mouse”, the prefix “h” represents “human”, and the suffix “sc” represents “scRNA-seq”. SRT, spatially resolved transcriptomics; scRNA-seq, single-cell RNA sequencing; AD, Alzheimer’s disease; BC, breast cancer; HD, high definition; DLPFC, dorsolateral prefrontal cortex; FFPE, formalin-fixed paraffin-embedded; MTG, middle temporal gyrus; PC, prefrontal cortex.

**Table 2:** List of benchmark methods

Method	Target data type	Core methodology	Implementation	Ref.	Task
scGeneClust	scRNA-seq	Splitting information-based maximum-spanning tree	Python package GeneClust (v0.0.1)	[11]	Identifying co-expressed genes
CS-CORE	scRNA-seq	Hierarchical clustering	Python package CS-CORE (v1.0.0)	[19]	Identifying co-expressed genes
COTAN	scRNA-seq	Hierarchical clustering	R package COTAN (v2.5.0)	[20]	Identifying co-expressed genes
CNN-PReg	SRT	CNN with graph regularization	https://github.com/kuanglab/CNN-PReg, commit 5a8505d (no package available)	[10]	Identifying co-expressed genes
Giotto	SRT	Hierarchical clustering	R package Giotto (v3.2)	[15]	Identifying co-expressed genes
STUtility	SRT	Non-negative matrix factorization	R package STUtility (v1.1.1)	[21]	Identifying co-expressed genes
SPARK	SRT	Hierarchical clustering	R package SPARK (v1.1.1)	[22]	Identifying co-expressed genes
CS-CORE	scRNA-seq	The joint distribution over UMI counts	Python package CS-CORE (v1.0.0)	[19]	Predicting gene interactions
COTAN	scRNA-seq	A statistic based on contingency tables of binarized UMI counts	R package COTAN (v2.5.0)	[20]	Predicting gene interactions
Giotto	SRT	Pearson correlation	R package Giotto (v3.2)	[15]	Predicting gene interactions
scGeneClust	scRNA-seq	Feature relevance	Python package GeneClust (v0.0.1)	[11]	Selecting feature genes
VST	scRNA-seq	Variances of standardized counts	Python package SCANPY (v1.9.3)	[16]	Selecting feature genes
M3Drop	scRNA-seq	Differences between gene-specific parameters and the global parameter	R package M3Drop (v1.12.0)	[17]	Selecting feature genes
SpatialDE	SRT	Likelihood ratio tests based on Gaussian process regression	Python package SpatialDE (v1.1.3)	[9]	Selecting feature genes
SPARK-X	SRT	Non-parametric covariance tests	R package SPARK (v1.1.1)	[22]	Selecting feature genes
BinSpect (kmeans)	SRT	Fisher tests on gene expression levels binarized with k-means	R package Giotto (v3.2)	[15]	Selecting feature genes
BinSpect (rank)	SRT	Fisher tests on gene expression levels binarized with threshold ranking	R package Giotto (v3.2)	[15]	Selecting feature genes

*Note*: CS-CORE, cell-type-specific co-expressions; COTAN, CO-expression Tables ANalysis; CNN, convolutional neural network; CNN-PReg, CNN with protein–protein interaction-graph regularization; SPARK, spatial pattern recognition via kernels; VST, variance stabilizing transformation; BinSpect, Binary Spatial Extraction; UMI, unique molecular identifier; M3Drop, fitting a Michaelis-Menten function to the relationship between mean expression and dropout-rate; SPARK-X, SPARK-eXpedited.

## Method

### Datasets

Our study involves 19 publicly available SRT datasets. In dataset names, the prefixes “m” and “h” represent “mouse” and “human”, respectively, while the suffix “sc” represents “scRNA-seq”. Three adult mouse brain datasets were downloaded from the 10X Genomics official website: mBrain-SRT (https://support.10xgenomics.com/spatial-gene-expression/datasets/1.1.0/V1_Adult_Mouse_Brain); mouse brain-Formalin-Fixed Paraffin-Embedded (mBrain-FFPE; https://www.10xgenomics.com/datasets/adult-mouse-brain-if-stained-ffpe-1-standard-1-3-0); and mouse brain-high definition (mBrain-HD; https://www.10xgenomics.com/datasets/visium-hd-cytassist-gene-expression-libraries-of-mouse-brain-he). Twelve slices of DLPFC (hDLPFC-SRT) datasets were acquired through spatialLIBD [23] (https://research.libd.org/spatialLIBD/). Three datasets from human middle temporal gyrus (MTG) of both Alzheimer’s disease (AD) patients and healthy individuals [hMTG-AD-SRT (sample 2-3), hMTG-health-SRT (sample 1-1), and hMTG-health-SRT2 (sample 144105)] were downloaded from the Gene Expression Omnibus (GEO: GSE220442 [12] and GSE226663 [24]). The breast cancer dataset (hBC-SRT) was downloaded from the 10X Genomics website (https://www.10xgenomics.com/datasets/human-breast-cancer-block-a-section-1-1-standard-1-1-0). We additionally paired our SRT datasets with six scRNA-seq/single-nucleus RNA sequencing (snRNA-seq) datasets. The mouse brain cortex dataset (mCortex-sc) [25] was downloaded from the Gene Expression Omnibus (GEO: GSE115746). The human brain cortex dataset (hCortex-sc) was obtained from the Allen Brain Atlas [26] and paired with hDLPFC-SRT datasets. From the hCortex-sc dataset, we extracted cells in MTG to form a new dataset (hMTG-health-sc), which was then paired with the hMTG-health-SRT dataset. Another MTG dataset from a healthy donor [hMTG-health-sc2 (sample HC14MTG)] was downloaded from the Gene Expression Omnibus (GEO: GSE188545 [27]) and paired with hMTG-health-SRT2. The hMTG-AD-sc dataset, also from the Allen Brain Atlas, was paired with the hMTG-AD-SRT dataset. The human prefrontal cortex-health-sc (sample NC3) [hPC-health-sc (sample NC3)] dataset, comprising cells from the prefrontal cortex of a healthy donor, was downloaded from the Gene Expression Omnibus (GEO: GSE157827 [28]) and paired with hDLPFC-SRT (slice 151673). The hBC-sc dataset (sample CID4471) was downloaded from the Gene Expression Omnibus (GEO: GSE176078 [29]) and paired with the hBC-SRT dataset.

### Data preprocessing

For both scRNA-seq and SRT data, we adhered to the standard pipeline of data preprocessing provided by the SCANPY package [30], which involves several steps. Firstly, we filtered out mitochondrial and External RNA Controls Consortium (ERCC) spike-in genes. Secondly, genes detected in fewer than 10 cells in scRNA-seq datasets or fewer than 10 spots in SRT datasets were excluded. Cells with fewer than 200 detected genes in scRNA-seq datasets were also removed. We did not perform filtering on spatial spots in SRT datasets to maintain spatial data integrity. Lastly, the gene expression counts of both scRNA-seq and SRT datasets were normalized by library size, followed by log-transformation.

### The algorithm of LEGEND

LEGEND’s framework is structured into two primary stages for cell type and tissue domain pseudo-labeling and gene clustering, as illustrated in Figure 1 and **Algorithms 1** and **2**. The core idea of LEGEND is that co-expressed and co-functional genes typically exhibit similar expression patterns in both scRNA-seq and SRT datasets, which can be identified through integrated gene clustering. In Stage I, LEGEND begins by generating highly confident (HC) pseudo-labels of spatial domains for SRT data, as detailed in the following section. The pseudo-labeling of single cells in scRNA-seq data is discussed in the “Pseudo-labeling of cells in unannotated scRNA-seq/snRNA-seq datasets” section in File S1. In Stage II, these pseudo-labels are utilized to estimate the similarity and discriminability of gene expression patterns across cell types and tissue domains in terms of relevance, redundancy, and complementarity from information theory (see “Gene relevance, redundancy, and complementarity” section below). Next, gene relationships are embedded in a gene redundancy graph, where nodes represent individual genes and edge weights indicate the redundancy between connected gene pairs. Leveraging a novel hierarchical clustering algorithm, LEGEND assigns informationally redundant genes to the same groups, while separating informationally complementary ones into distinct groups to maximize intra-group redundancy and inter-group complementarity. As such, these groups represent functionally distinct gene modules, each comprising genes that are co-expressed and co-functional across cell types and tissue domains.

**Algorithm 1:** LEGEND stage I — pseudo labeling of spatial spots in SRT data

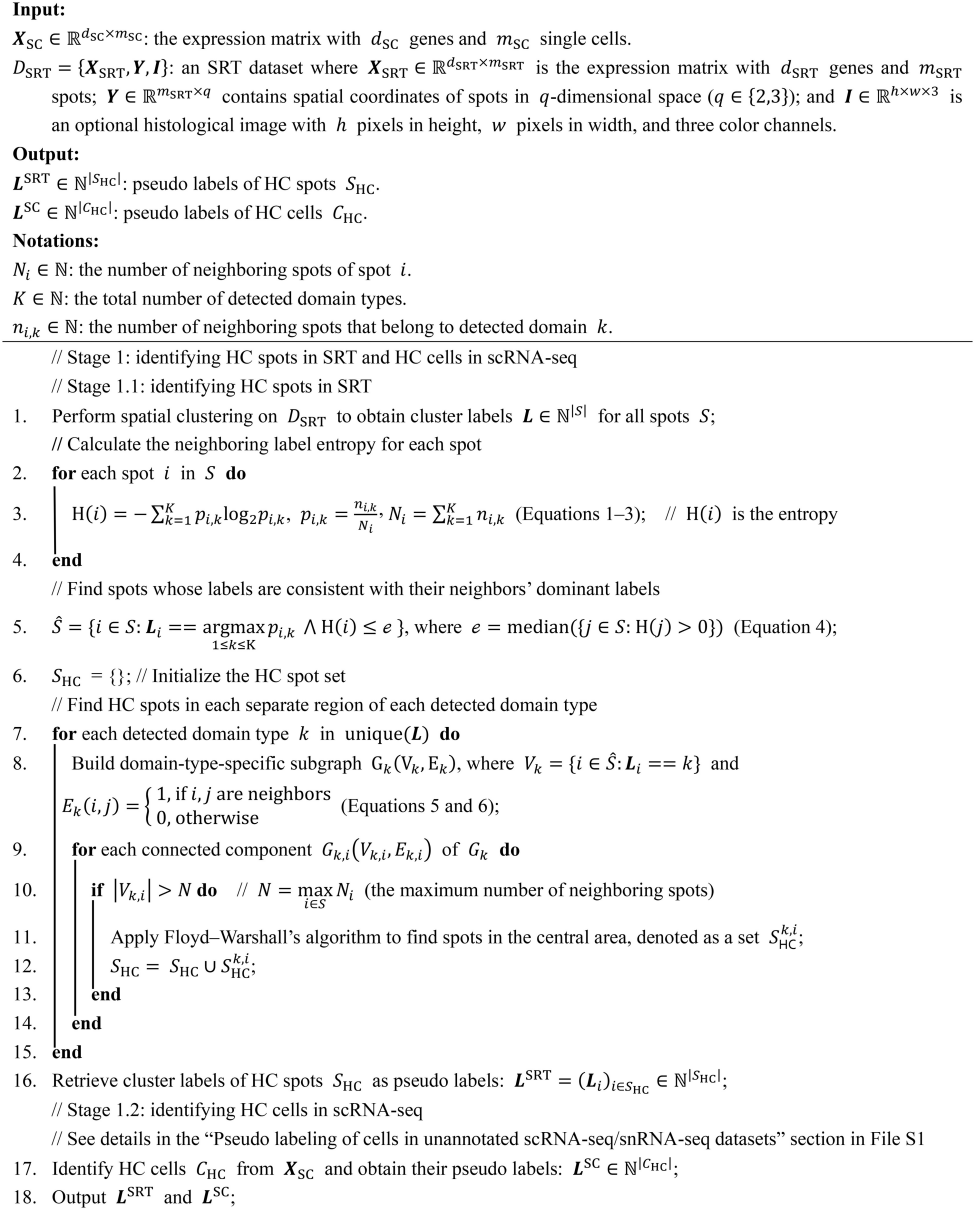

**Algorithm 2:** LEGEND stage II — hierarchical gene clustering based on the gene redundancy graph

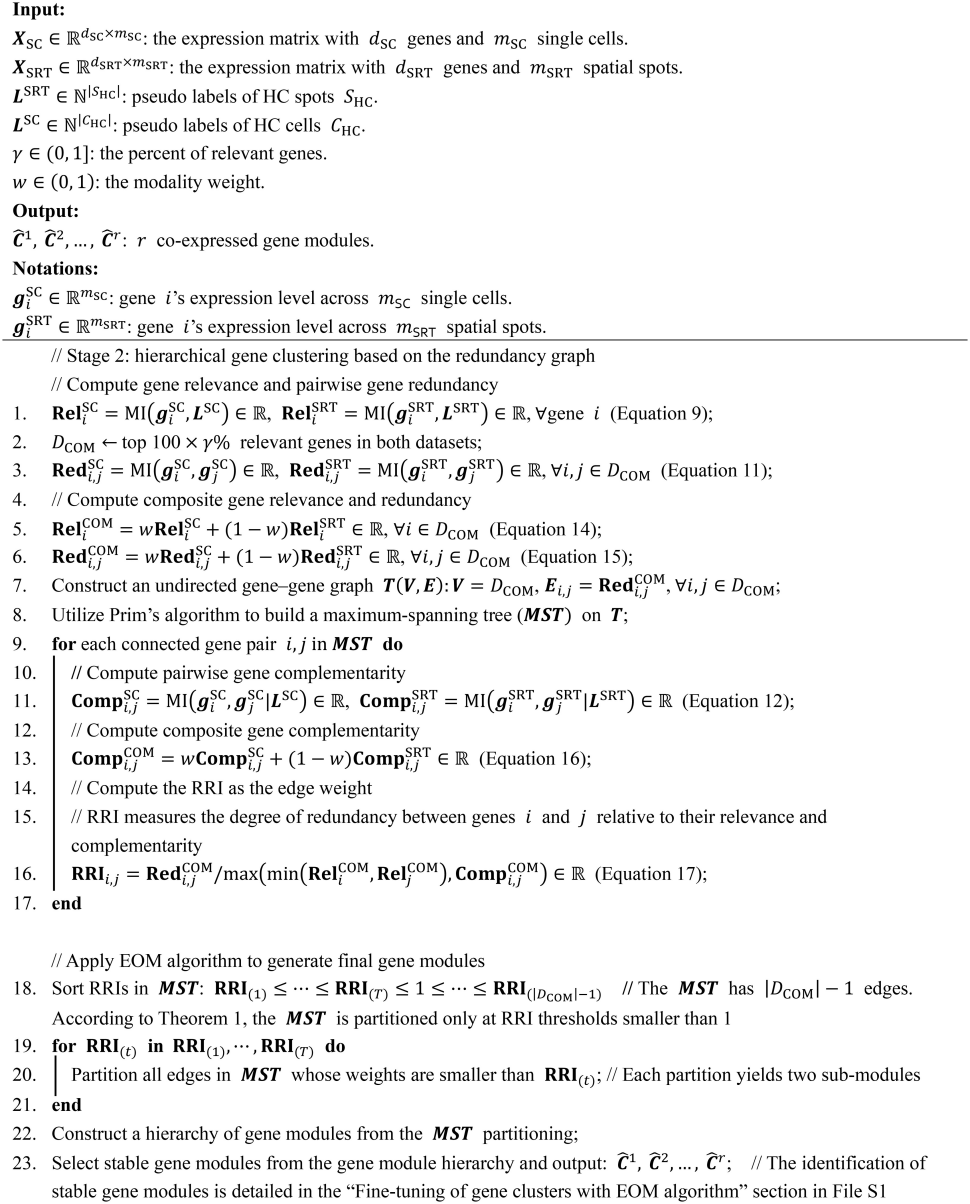

### Pseudo-labeling of spatial spots in SRT data

In this stage, a pseudo-semi-supervised approach is adopted to acquire pseudo-labels for spatial spots that are likely to be of the same domain types, termed HC spots. Stage I unfolds in two steps (Figure 1A). Initially, spatial spots are clustered into spatial domains using an off-the-shelf spatial clustering method, such as SpaGCN, from which HC spots are selected. LEGEND does not rely on a highly accurate spatial clustering method. As long as the method achieves sufficient accuracy, LEGEND can effectively identify HC spots within spatial clusters (Figure S1). Since there is greater confidence in the cluster labels of spots at the center of large, homogeneous spatial domains, peripheral spots are excluded upfront. A spot is deemed peripheral if its neighboring spot labels are significantly different from its label or show considerable heterogeneity. Therefore, a spot i is excluded if the labels of its neighboring spots are predominantly different or if the label distribution exhibits high entropy, which is defined as:


(1) $$H(i)=-\sum_{k=1}^{K}p_{i,k}\log_{2}p_{i,k}$$

(2) $$p_{i,k}=\frac{n_{i,k}}{N_{i}}$$

(3) $$\sum_{k=1}^{K}n_{i,k}=N_{i}$$

where $N_{i}$ is the number of neighboring spots of spot i, K is the total number of domain types, $n_{i,k}$ is the number of neighboring spots that belong to domain type k ∈ {1, ⋯, K}, and $p_{i,k}$ is the proportion of neighboring spots that belong to domain type k. Subsequently, we construct a graph $G_{k}(V_{k},\;E_{k})$ for each domain type k:


(4) $$\hat{S}=\{i\;:\;H(i)\;\leq \;e,\;i\;\in \;S\},\;e=\mathrm{median}(\{H(j)>0\;|\;j\;\in \;S\})$$

(5) $$V_{k}=\{i\;:\;L(i)\;==\;k,\;i\;\in \;\hat{S}\}$$

(6) $$E_{k}(i,\;j)=\{\begin{array}{l} \;1,\;\mathrm{if}\;i,\;j\;\mathrm{are}\;\mathrm{neighbors}\\ \;0,\;\mathrm{otherwise} \end{array}$$

where S denotes the set of all spots, $\hat{S}$ denotes the set of spots after entropy filtering, L(i) denotes the domain type label of spot i. Note that spots of the same domain type may locate in separated spatial regions, so the graph of a domain type may consist of multiple disconnected subgraphs. We exclude subgraphs whose sizes are smaller than the number of neighboring tiles (*e.g.*, 6 for 10X Visium data). To identify HC spots, we first need to pinpoint the graph center for each remaining subgraph. Specifically, for a given spot u within a subgraph $G_{k,i}(V_{k,i},\;E_{k,i})\;\in \;G_{k}(V_{k},{\;E}_{k})$, its eccentricity is computed as $\varepsilon_{u}=\mathrm{ma}x_{v\;\in {\;V}_{k,i}}(d_{u,v}),\;\forall \;v\;\in \;V_{k,i}$. Here $d_{u,v}$ denotes the distance between spots u and v, which is calculated using the Floyd–Warshall’s algorithm [31]. The center $\psi (G_{k,i})$ and radius $r(G_{k,i})$ of $G_{k,i}(V_{k,i},\;E_{k,i})$ are:


(7) $$r(G_{k,i})=\mathrm{mi}n_{u\;\in {\;V}_{k,i}}\varepsilon_{u}$$

(8) $$\psi (G_{k,i})=\{v\;\in \;V_{k,i}\;:\;\varepsilon_{v}=r(G_{k,i})\}$$

Spots whose distance to $\psi (G_{k,i})$ is less than 0.6 $\times \;r(G_{k,i})$ are deemed as close to the graph center and selected as HC spots of $G_{k,i}$.

### Gene relevance, redundancy, and complementarity

Gene relevance, redundancy, and complementarity are computed as mutual information (MI) between gene–spot/cell label pairs or gene–gene pairs. Given that MI is originally defined for discrete variables and gene expression levels are continuous, we leverage a *k*-nearest neighbor-based algorithm (File S1, see the “Calculation of mutual information involving continuous variables” section for details) when MI calculation involves continuous variables.

#### Gene relevance

The relevance of a gene reflects its discriminative power on different domain/cell type labels. Let $g_{i}\in {\mathbb{R}}^{n}$ denote gene i’s expression level across spatial spots or cells, $H(g_{i})$ denote its entropy, and $H(g_{i}\;|\;L)$ denote its conditional entropy given the labels. Gene i’s relevance is computed as:


(9) $$\begin{array}{l} R\mathrm{el}(g_{i})=\mathrm{MI}(g_{i},\;L)=H(g_{i})-H(g_{i}\;|\;L) \end{array}$$

We further define the conditional relevance of gene i given $g_{j}\;$as:


(10) $$\mathrm{Rel}(g_{i}\;|{\;g}_{j})=\mathrm{MI}(g_{i},\;L\;|{\;g}_{j})=H(g_{i}\;|{\;g}_{j})-H(g_{i}\;|{\;g}_{j},\;L)\;=\;H(g_{j}\;|\;g_{i})-H(g_{j}\;|\;g_{i},\;L)$$

where $H(g_{i}\;|{\;g}_{j})$ and $H(g_{i}\;|{\;g}_{j},\;L)$ are conditional entropies.

#### Gene redundancy

The redundancy between a pair of genes i and j is defined as the MI between their expression levels:


(11) $$\begin{array}{ll} R\mathrm{ed}(g_{i},\;g_{j})=\mathrm{MI}(g_{i},\;g_{j})=H(g_{i})-H(g_{i}\;|\;g_{j})\; & \end{array}$$

$$=\;H(g_{j})-H(g_{j}\;|\;g_{i})$$

Gene redundancy represents the amount of information shared between genes, implying that discarding highly redundant genes has minimal impact on the overall information and enhances the informational efficiency of the feature gene set.

#### Gene complementarity

The complementarity between genes i and j measures the additional information gained for label discrimination when both genes are considered together compared to each on its own:


(12) $$\mathrm{Comp}\begin{array}{ll} \left(g_{i},\;g_{j})=\mathrm{MI}(g_{i},\;g_{j}\;|\;L)=H(g_{i}\;|\;L)-H(g_{i}\;|{\;g}_{j},\;L\right) & \end{array}$$

$$=\;H(g_{j}\;|\;L)-H(g_{j}\;|\;g_{i},\;L)$$

Furthermore, using the notation defined previously, we have the following Theorem 1 (Equation 13; see the “Proof of Theorem 1” section in File S1 for details):


(13) $$\mathrm{Red}(g_{i},\;g_{j})<\mathrm{Comp}(g_{i},{\;g}_{j})\;⟺\;\mathrm{Rel}(g_{i})<\mathrm{Rel}(g_{i}\;|\;g_{j})\;\&\;\mathrm{Rel}(g_{j})<\;\mathrm{Rel}(g_{j}\;|\;g_{i})$$

It shows that if the complementarity of two genes is larger than their redundancy, the relevance of one gene can be enhanced given the expression levels of the other, *i.e.*, $\mathrm{Rel}(g_{i}\;|\;g_{j})>\mathrm{Rel}(g_{i})$.

### Redundancy graph-based hierarchical gene clustering

Gene relevance, redundancy, and complementarity in the SRT and scRNA-seq datasets are computed using the labels of HC spots and single cells. Only the most informative genes (*i.e.*, the top 80% in relevance in both datasets) are retained. For two given genes i and j, their composite gene relevance, redundancy, and complementarity (RelCOM, RedCOM, and $\mathrm{Com}p^{\mathrm{COM}}$) are computed as weighted averages of the individual metrics derived from the SRT and scRNA-seq datasets:


(14) RelCOM(gi)=wRelSC(gi) + (1-w)RelSRT(gi)

(15) RedCOM(gi,gj)=wRedSC(gi,gj)+(1-w)RedSRT(gi,gj)

(16) $$\mathrm{Com}p^{\mathrm{COM}}(g_{i},g_{j})=w\mathrm{Com}p^{\mathrm{SC}}(g_{i},g_{j})+(1-w)C\mathrm{om}p^{\mathrm{SRT}}(g_{i},g_{j})$$

where w ∈ (0,1) is the weight factor for balancing the contribution of SRT and scRNA-seq data. Following this, an undirected complete gene graph is constructed, wherein vertices represent genes and edge weights equal the composite redundancies between connected gene pairs. The graph is partitioned into subgraphs representing gene clusters, aiming to maximize intra-cluster gene redundancy and inter-cluster complementarity. Instead of partitioning the complete graph, a nondeterministic polynomial-time hard (NP-hard) problem, we conduct the partition on a maximum spanning tree (MST) extracted from the graph using Prim’s algorithm [32]. This MST not only significantly reduces the number of edges to be partitioned but also preserves the graph’s most representative redundancy structure. Therefore, gene clusters separated from this MST exhibit maximized intra-cluster redundancy. Formally, we define the relative redundancy index (RRI) of the edge $e_{i,j}$ connecting two neighboring genes i and j on the MST as:


(17) RRI(ei,j)=RedCOM(gi,gj)max(min(RelCOM(gi),RelCOM(gj)),CompCOM(gi,gj))

The Excess of Mass (EOM) algorithm [33] is employed to partition the MST at different RRI thresholds: $e_{i,j}$ is preserved only if $\mathrm{RRI}(e_{i,j})\;$is above the current RRI threshold, generating a hierarchy of gene clusters. As the RRI threshold increases along the hierarchy in the top-down direction, fewer edges are preserved, leading to the formation of finer gene clusters toward the hierarchy’s bottom. Among all clusters within the hierarchy, clusters of high quality and sensible size are automatically determined based on their stability coefficients, which essentially measure their “persistence” along the hierarchy (see the “Fine-tuning of gene clusters with EOM algorithm” section in File S1 for details). The maximum RRI threshold is set to 1 so that genes *i* and *j* are assigned to different clusters only if either of the following two equations is satisfied:


(18) RedCOM(gi,gj)<CompCOM(gi,gj)

(19) RedCOM(gi,gj)<min(RelCOM(gi), RelCOM(gj))

Equation 18 implies that two genes with greater complementarity than their redundancy should be separated into different clusters to enhance their overall relevance, as suggested by Theorem 1 above. Equation 19 implies that two genes should not be in the same cluster if they are not sufficiently redundant relative to their minimum relevance. These two conditions ensure the informational complementarity between different clusters. Furthermore, clusters with sizes smaller than 5 are considered as noise groups of genes “falling out of their parent cluster” rather than “real clusters”, and are merged back into their parent clusters in the hierarchy.

### Biological analysis

We investigated the biological significance of LEGEND-generated gene clusters through a case study involving two pairs of SRT and scRNA-seq datasets from the MTG of both AD patients and healthy individuals. The analysis proceeded with the following steps.

#### Selection of top relevant gene clusters

Gene clusters were sorted in descending order by the maximum RelCOM of their component genes, from which top-ranked clusters were selected until they collectively encompassed 100 genes. Gene clusters selected from the AD patient datasets constituted the disease group, while those from the healthy individual datasets formed the normal group. Both groups were subjected to enrichment and co-function analyses. Concurrently, an equal number of genes were randomly selected as a control group.

#### Enrichment analysis

BPs enriched in the given gene set can be identified via enrichment analysis. We conducted both Gene Ontology (GO) and Kyoto Encyclopedia of Genes and Genomes (KEGG) pathway enrichment analyses for disease, normal, and control gene groups to assess their biological relevance to MTG functions and AD pathology. The R package clusterProfiler (v4.2.2) [34] was utilized to identify Gene Ontology biological processes (GOBPs) or KEGG pathways enriched in the genes of the three groups. Enrichment resultant *P* values were adjusted via the Benjamini–Hochberg procedure, and the 20 most significantly enriched GOBPs/KEGG pathways were selected for further biological analyses, as described in Results.

#### Gene co-function analysis

Functional coherence of a gene set within a GOBP or KEGG pathway can be assessed using the neighbor-voting method proposed by Ballouz and his colleagues [35]. The rationale is that if some genes’ involvement in GOBPs/pathways can be more accurately predicted by other genes, then these genes exhibit greater functional coherence. Formally, let d denote the number of genes, q denote the number of GOBPs/pathways, $\Sigma \in R^{d\times d}$ denote the gene–gene similarity matrix of the SRT dataset, $S\in R^{d\times d}$ denote the gene–gene similarity matrix of the scRNA-seq dataset, and $A\in R^{d\times q}$ denote the gene annotation matrix. Then we have:


(20) $$S\;=\;-\frac{1}{2}(I\;-\;\frac{M}{d}){\left|\Delta^{\mathrm{pearson}}\right|}_{L1}(I\;-\;\frac{M}{d})$$

(21) $$\Sigma \;=\;-\frac{1}{2}(I\;-\;\frac{M}{d})\Delta^{\mathrm{euclid}}(I\;-\;\frac{M}{d})$$

(22) S = wΣ + (1 - w)S

(23) Aij={ 1, if gene i is involved in the j-th GOBP/pathway 0, otherwise

where $M\;\in {\;R}^{d\times d}$ is a matrix of 1s, and $\Delta^{\mathrm{pearson}}\;\in \;{\mathbb{R}}^{d\times d}$ and $\Delta^{\mathrm{euclid}}\;\in \;R^{d\times d}\;$are gene–gene distance matrices for the scRNA-seq and SRT datasets, as calculated from Equations 32 and 33, respectively (see “Evaluation metrics” section). $S\in R^{d\times d}$ is a weighted average of Σ and S, with weight w assigned to Σ.

The prediction accuracy is assessed via three-fold cross-validation conducted on the top 20 enriched GOBPs/pathways. In each fold, one third of the genes are randomly selected as the test dataset, and their associated GOBPs/pathways are masked by setting the corresponding rows in A to zeros. Given a gene t in the test dataset, its predicted probability of being involved in the j-th GOBP/pathway is calculated as:


(24) $$p_{tj}=\sum_{i=1}^{d}S_{ti}A_{ij}\;/\;\sum_{i=1}^{d}S_{ti}$$

The prediction results are evaluated using the area under the receiver operating characteristic curve (AUC) metric, where a higher value indicates greater functional coherence among the genes in that GOBP/pathway. Genes involved in the selected GOBPs and KEGG pathways were obtained using the R package AnnotationDbi (v1.56.2) and KEGGREST (v1.34.0), respectively. The R package Extending “Guilt by Association” by Degree (EGAD; v1.22.0) was employed to conduct the neighbor-voting-based prediction.

### Identifying disease-associated gene crosstalk

Given an scRNA-seq dataset $X_{1}\;\in \;{\mathbb{R}}^{N_{1}\times G}$ and an SRT dataset $X_{2}\;\in \;R^{N_{2}\times G}$, where $N_{1}$ denotes the number of single cells, $N_{2}$ denotes the number of spots, and G denotes the number of genes, we first calculate its combined Pearson correlation matrix of genes $\Theta \in {\mathbb{R}}^{G\times G}:=w\times \mathrm{corr}(X_{1})+(1-w)\times \mathrm{corr}(X_{2})$. Let $\Sigma \;\in \;R^{G\times G}$ denote the gene–gene redundancy matrix generated by LEGEND. Σ is used as a surrogate non-parametric covariance matrix in a Gaussian copula graphical model (GCGM) to estimate the sparsified gene–gene precision matrix $\hat{\Lambda \;}\in {\;R}^{G\times G}$:


(25) $$\hat{\Sigma }=\Sigma \;⊙\;\mathrm{sign}(\Theta )$$

(26) $$\hat{\Lambda }=\underset{\Lambda \in R_{++}^{G}}{\mathrm{argmin}}-\;\log\left|\Lambda \right|\;+\;\mathrm{tr}(\hat{\Sigma }\Lambda )\;+\;\alpha {\left\|\Lambda \right\|}_{1}\;$$

where ⊙ is the Hadamard product. $\hat{\Lambda }$ is then converted into the gene partial correlation matrix $\Phi \;\in {\;R}^{G\times G}$, wherein nonzero values indicate plausible gene–gene interactions:


(27) $${\hat{\Phi }}_{i,j}=\frac{\Lambda_{i,j}}{\sqrt{\Lambda_{i,i}\Lambda_{j,j}}+\varepsilon },\;\forall i,j\in [1,N]$$

where ε =1E−6 is for numerical stability.

### Selecting an informationally optimal set of feature genes for cell/spot clustering

Here, we aim to select an informationally optimal set of feature genes, *i.e.*, those with the most relevance and complementarity and the least redundancy. To maximize complementarity, we keep all gene clusters as they represent functionally complementary gene modules. Meanwhile, as genes within the same gene cluster are highly redundant, we only select the top relevant genes from each cluster as representative genes to maximize relevance while minimizing redundancy. Formally, given the k-th gene cluster ${\hat{C}}^{k}$, its representative genes are selected as:


(28) Rk={j | RelCOM(gj) ≥ P80k, j ∈ C^k}

where P80k=percentile({RelCOM(gi) | i ∈ C^k}, 80) denotes the genes that are more relevant than 80% of genes in the cluster. The rationale behind determining 80% as the relevance threshold is detailed in the “Impact of the number of selected feature genes on single-cell/spatial clustering performance” section in File S1, and illustrated in Figures S2 and S3. Finally, the union of representative genes across all clusters (*i.e.*, ∪${\;}_{k=1}^{r}R_{k},\;r$ is the total number of gene clusters) are employed as the informationally optimal set of feature genes for downstream spatial and single-cell clustering.

### Evaluation metrics

#### Gene clustering

Davies–Bouldin (DB) index [36] is used to assess the performance of gene clustering in SRT and scRNA-seq datasets:


(29) $$\mathrm{DB}=\frac{1}{n}\sum_{i=1}^{n}\underset{i\neq j}{\max}\frac{d_{i}\;+\;d_{j}}{d_{\left(i,j\right)}}$$

(30) $$d_{i}=\frac{2}{n_{i}(n_{i}-1)}\sum_{p,q\in G_{i},p\neq q}\delta_{p,q}$$

(31) $$d_{\left(i,j\right)}=\delta_{c_{i},c_{j}}$$

where $\delta_{p,q}$ denotes the distance between genes p and q, n denotes the number of clusters, $d_{i}$ is the intra-cluster distance of cluster i (calculated as the average distance between genes of cluster i), and $d_{\left(i,j\right)}$ is the inter-cluster distance between clusters i and j (calculated as the distance between cluster centroids $c_{i}\;$and $c_{j}$). DB index represents the ratio of intra-cluster distance (compactness) to inter-cluster distance (separateness), so a smaller value of DB index indicates better clustering performance. To evaluate the co-expression of gene clusters in the scRNA-seq dataset and the spatial coherence of gene clusters in the SRT dataset, Pearson distance and Euclidean distance are used in the calculation of the DB index, respectively [10]:


(32) $$\delta_{p,q}^{\mathrm{pearson}}=1-\rho_{p,q}$$

(33) $$\delta_{p,q}^{\mathrm{euclid}}=\sqrt{{\left(x_{p}-x_{q}\right)}^{T}W(x_{p}-x_{q})},\;W\in R^{N_{x}N_{y}\times N_{x}N_{y}}$$

where $\rho_{p,q}$ represents the Pearson correlation between genes p and q in the scRNA-seq dataset. $N_{x}$ and $N_{y}\;$ denote the number of spatial spots along the horizontal and vertical axes of the spatial map of the SRT dataset, respectively. $x_{p}\;$ is the flattened expression matrix of gene  p in the SRT dataset, and  W is the weight matrix calculated based on the spatial spots’ locations using a Gaussian kernel, reflecting the spatial closeness of the spatial spots.

#### Spatial/single-cell clustering

The accuracy of clustering for spatial spots/cells is assessed using the Adjusted Rand Index (ARI) and Normalized Mutual Information (NMI). Let n denote the total number of spots/cells, $n_{ij}$ denote the number of spots/cells of domain/cell type i in cluster j, $a_{i}$ denote the total number of spots/cells of domain/cell type i, and $b_{j}$ denote the number of all spots/cells in cluster j. Additionally, L refers to the true labels of spots/cells, and $\tilde{L}$ refers to the cluster labels of spots/cells. The ARI and NMI are defined as follows:


(34) $$\mathrm{ARI}=\frac{\sum_{ij}\left(\begin{array}{l} n_{ij}\\ 2 \end{array}\right)-[\sum_{i}\left(\begin{array}{l} a_{i}\\ 2 \end{array}\right)\sum_{j}\left(\begin{array}{l} b_{j}\\ 2 \end{array}\right)]/(\begin{array}{l} n\\ 2 \end{array})}{\frac{1}{2}[\sum_{i}\left(\begin{array}{l} a_{i}\\ 2 \end{array}\right)+\sum_{j}\left(\begin{array}{l} b_{j}\\ 2 \end{array}\right)]-[\sum_{i}\left(\begin{array}{l} a_{i}\\ 2 \end{array}\right)\sum_{j}\left(\begin{array}{l} b_{j}\\ 2 \end{array}\right)]/(\begin{array}{l} n\\ 2 \end{array})}$$

(35) $$\mathrm{NMI}=\frac{2\times \;\mathrm{MI}(\tilde{L},L)}{H(\tilde{L})+H(L)}$$

where MI represents MI, and H represents entropy.

## Results

### LEGEND effectively identifies cross-modal clusters of co-expressed genes

We compared the clustering performance of LEGEND against six state-of-the-art competing methods (CNN-PReg, Giotto, CS-CORE, COTAN, SPARK, and STUtility) in identifying co-expressed gene clusters. We also included scGeneClust and LEGEND-no-sc, both of which use scRNA-seq data only, as competing methods. Our analysis involved 12 human DLPFC SRT datasets (hDLPFC-SRT), all paired with an scRNA-seq dataset (hCortex-sc) from multiple human cortical areas; three mouse brain SRT datasets (mBrain-SRT, mBrain-FFPE, and mBrain-HD), accompanied by a corresponding scRNA-seq dataset (mCortex-sc); one SRT dataset (hMTG-health-SRT) and its matching scRNA-seq dataset (hMTG-health-sc), both sampled from the healthy MTG; one SRT dataset (hBC-SRT) paired with an scRNA-seq dataset (hBC-sc) from breast cancer patients; and one SRT dataset (hMTG-AD-SRT) coupled with an scRNA-seq dataset (hMTG-AD-sc) from the MTG of AD patients (Table 1). This compilation resulted in a total of 18 pairs of SRT and scRNA-seq datasets.

LEGEND performed integrated gene clustering on each dataset pair. The resulting clusters were assessed in three aspects: (1) the intra-cluster co-expression, proxied by the similarity between spatial expression patterns of genes within the same cluster, (2) the co-expression of gene clusters across cell types, and (3) the spatial coherence of gene clusters across tissue domains. Although the true number of gene clusters cannot be determined, clustering performance can still be evaluated since it would show sensible clustering results only if the clustering algorithm works as expected. To control the gene cluster size, we set the target number of clusters to 500 for all tested methods, so that on average each cluster has approximately 40 genes, approximating the average number of genes in a typical KEGG pathway [37].

**Figure 2:**
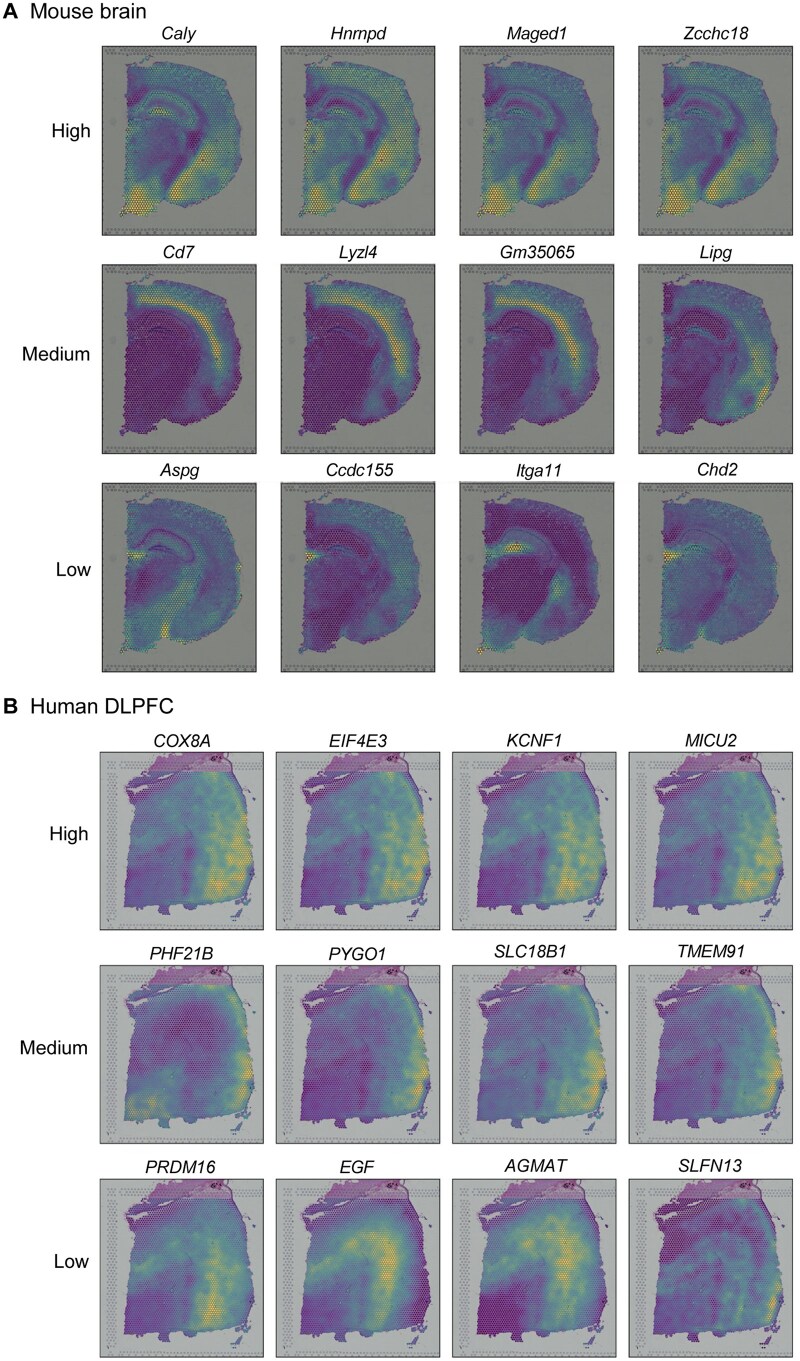
Spatial expression patterns of LEGEND-identified gene clusters in mouse brain and human DLPFC Gene clusters identified by LEGEND from the SRT datasets of mouse brain and human DLPFC are divided into groups of high, medium, and low co-expression quality based on their ICQ values. Rows display a gene cluster randomly selected from each of these groups, respectively. The denoised spatial expression patterns of four genes randomly chosen from the cluster are visualized in each row. **A**. Mouse brain (the mBrain-SRT dataset). **B**. Human DLPFC (the hDLPFC-SRT dataset). ICQ, intra-cluster co-expression quality; DLPFC, dorsolateral prefrontal cortex.

To quantify the similarity of spatial expression patterns between genes within the same cluster, we defined an intra-cluster co-expression quality (ICQ) metric (File S1, see the “Intra-cluster co-expression quality metric” section for details). Briefly, we employed two computer vision techniques, Canny Edge Detector and Patch Brightness Detector, to extract and encode edge and brightness traits from each gene’s spatial expression map. The ICQ metric of a cluster was calculated as the average Pearson correlation coefficient between the trait encodings of its component genes. We further categorized the gene clusters in each dataset into three groups based on ICQ: high, medium, and low. Gene clusters with ICQ in the top third quantile range were assigned to the high-quality group, those in the bottom third quantile range were assigned to the low-quality group, and all remaining clusters were assigned to the medium-quality group. Next, we randomly selected a cluster from each of the three groups and then randomly chose four genes from each of the selected clusters. The denoised co-expression patterns of these chosen genes were plotted for comparison. As shown in **Figure 2**A and B, Clusters 1, 2, and 3 were selected from high, medium, and low ICQ groups, respectively, in the mBrain-SRT dataset and hDLPFC-SRT dataset (slice 151673). For both datasets, genes within each cluster exhibited similar spatial expression patterns, particularly for Clusters 1 and 2, demonstrating the effectiveness of LEGEND in identifying co-expressed genes. Similar results were observed across the mBrain-FFPE, mBrain-HD, and hBC-SRT datasets (Figure S4).

**Figure 3:**
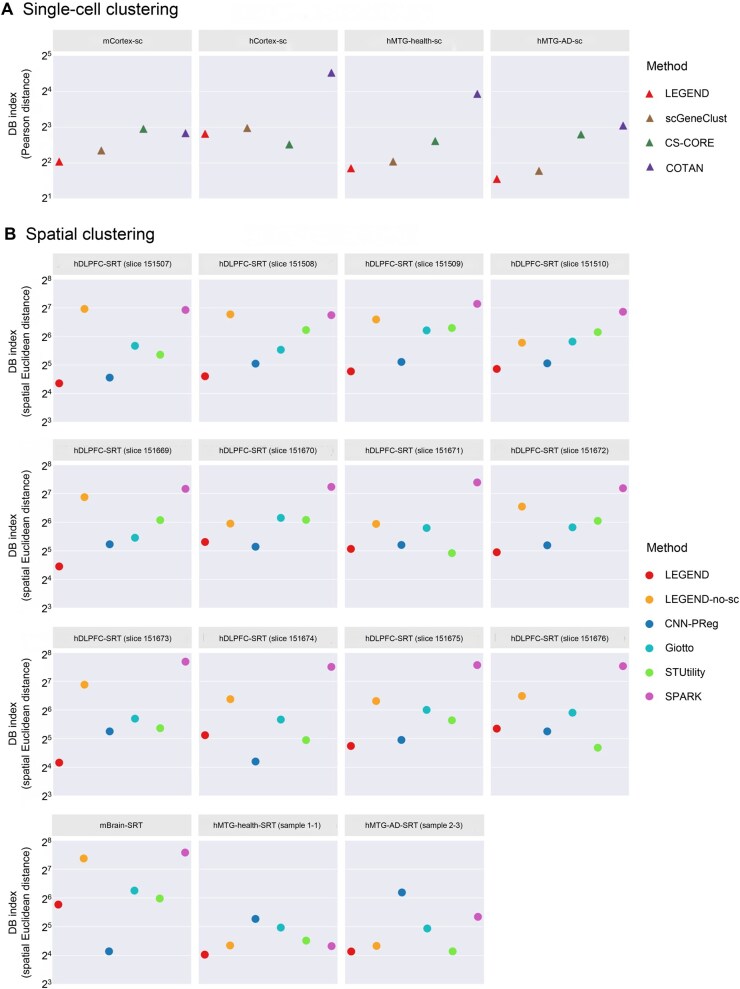
Performance comparison between LEGEND and competing methods for identifying gene co-expression groups in scRNA-seq and SRT datasets from mouse and human brain **A**. The overall quality of gene co-expression groups identified in each of the four scRNA-seq datasets is quantified using co-expression DB index (Y-axis), where a lower score indicates superior clustering quality. **B**. The overall quality of gene co-expression groups identified in each of the 15 SRT datasets is quantified using spatial coherence DB index (Y-axis), where a lower score indicates more effective clustering. In dataset names, the prefix “m” represents “mouse”, the prefix “h” represents “human”, and the suffix “sc” represents “scRNA-seq”. DB, Davies–Bouldin; AD, Alzheimer’s disease; MTG, middle temporal gyrus; CS-CORE, cell-type-specific co-expressions; COTAN, CO-expression Tables ANalysis; CNN-PReg, convolutional neural network with protein–protein interaction-graph regularization; SPARK, spatial pattern recognition via kernels.

The DB indices with Pearson distance and weighted Euclidean distance served as measures for evaluating the overall co-expression and spatial coherence of gene clusters, respectively (see “Evaluation metrics” section). Compared to the benchmark methods, LEGEND consistently generated gene clusters with lower values in both types of DB indices (**Figure 3**A and B, Figure S5) across scRNA-seq and SRT datasets, implying that LEGEND excels in grouping similar genes while separating dissimilar ones into distinct clusters at both cell type and tissue domain levels, which contributes to a more accurate identification of co-expressed and co-functional gene modules. Moreover, LEGEND’s superior performance over LEGEND-no-sc indicates that integrating scRNA-seq**/**snRNA-seq with SRT data can effectively enhance gene clustering.

### Biological analyses of LEGEND-generated gene clusters

We conducted GOBP enrichment, KEGG pathway enrichment, and gene co-function analyses on genes within clusters generated by LEGEND to evaluate their biological relevance to the dataset under investigation. This study involved two groups: a disease group, comprising an scRNA-seq dataset (hMTG-AD-sc) and an SRT dataset (hMTG-AD-SRT) from human MTG of AD patients, and a normal group, comprising an scRNA-seq dataset (hMTG-health-sc) and an SRT dataset (hMTG-health-SRT) from healthy human MTG. The biological analyses were performed on genes from the top relevant gene clusters in both groups (see “Selection of top relevant gene clusters” section) and a control group with an equal number of randomly selected genes. GOBPs and KEGG pathways were defined as AD-related or brain-related based on their connections to the relevant BPs (File S1, see the “Defining AD-related and brain-related GOBPs and KEGG pathways” section for details).

**Figure 4:**
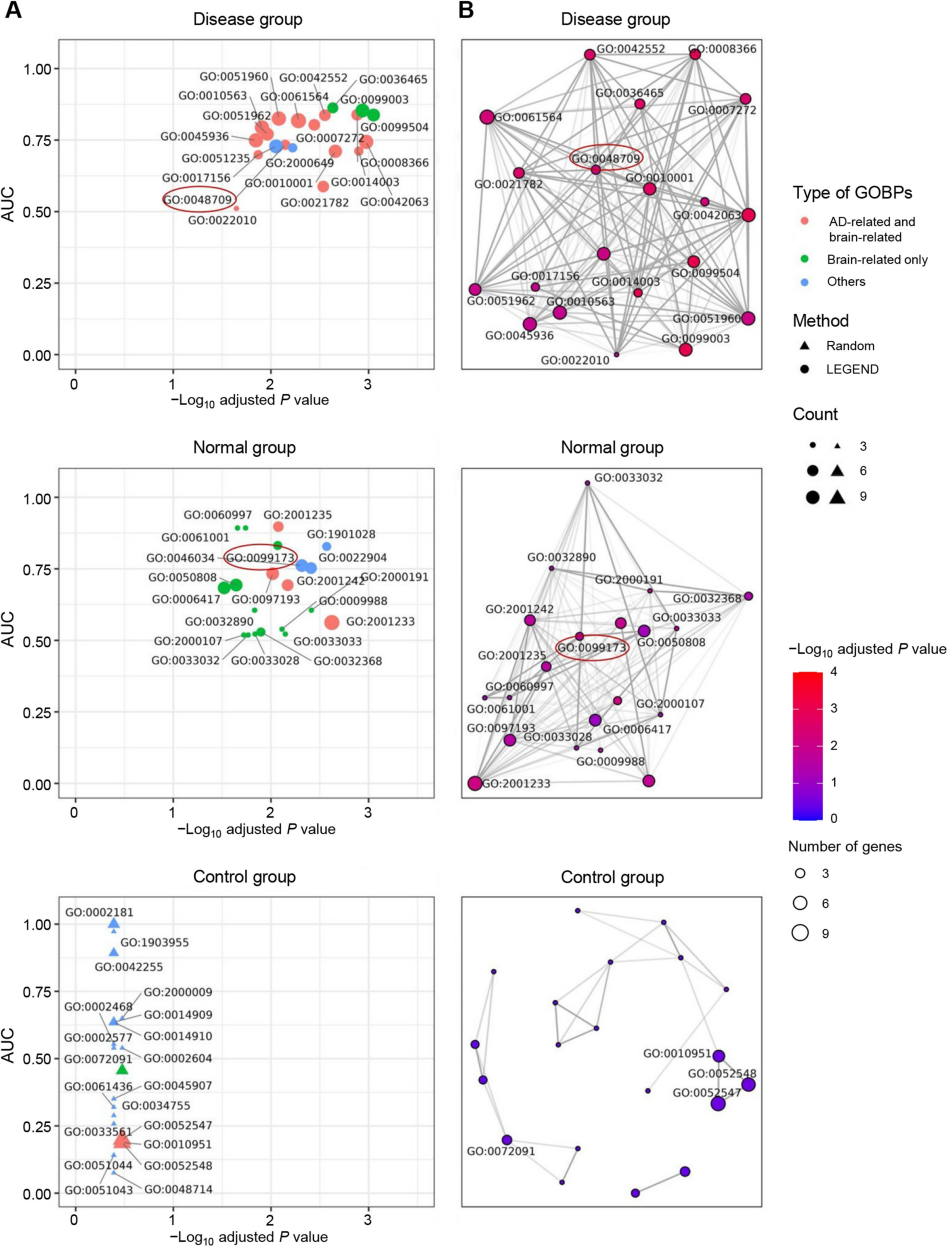
GOBP enrichment and gene co-function analyses of LEGEND-identified gene clusters Top relevant gene clusters collected from the datasets of MTG in both healthy and AD individuals form the normal and disease groups, respectively. A control group comprising an equal number of randomly selected genes serves as a benchmark. **A**. Top 20 significantly enriched GOBPs for each group. The X-axis shows the enrichment significance (−log_10_ adjusted *P* value). The Y-axis shows the AUC scores of top 20 GOBPs in the gene co-function analysis, where a higher score indicates stronger co-functionality within the BP. Red indicates AD-related and brain-related GOBPs, green indicates brain-related only GOBPs, and blue indicates other GOBPs. **B**. Enrichment maps of top 20 significantly enriched pathways for each group. Edge connectivity of the network indicates functional associations among GOBPs. Node color represents GOBP’s enrichment significance (−log_10_ adjusted *P* value), with red indicating higher significance. Node size denotes the number of genes involved in the BP. BP, biological process; GOBP, Gene Ontology biological process; AUC, area under the receiver operating characteristic curve.

Here, we only elaborated on the results of GOBP enrichment analysis. KEGG pathway enrichment analysis yielded similar results (Figure S6; Table S1). As shown in **Figure 4**A and Table S2, the adjusted *P* values of the top 20 significantly enriched GOBPs of the disease and normal groups were markedly lower than those in the control group, indicating that genes in the disease and normal groups are more likely to participate in the same GOBPs. Additionally, the disease group contained more AD-related GOBPs (15 GOBPs) than the normal (4 GOBPs) and control (2 GOBPs) groups, while the normal group had more brain-related GOBPs (13 GOBPs) than the control group (1 GOBP). These findings demonstrate LEGEND’s capability to identify biologically relevant gene groups within the dataset.

In the gene co-function analysis, functional coherence among genes within a GOBP was assessed by measuring the accuracy of predicting genes’ involvement in that GOBP using their neighboring genes (see “Gene co-function analysis” section). As shown in Figure 4A, the top 20 significantly enriched GOBPs in both disease and normal groups exhibited notably higher AUC values than those in the control group, supporting that genes in the first two groups tend to be co-functional within the same GOBPs. Additionally, as illustrated in Figure 4B, the GOBPs enriched in the disease group formed a single, densely connected network centered around co-functional elements implicated in the etiology of AD (*i.e.*, “oligodendrocyte differentiation, GO:0048709”), while the GOBPs enriched in the normal group were closely interconnected around co-functional elements related to neural and brain functions (*i.e.*, “postsynapse organization, GO:0099173”). In contrast, the GOBPs enriched in the control group formed several much smaller, loosely connected networks, most of which lacked co-functionality and showed no relevance to AD or brain function. Collectively, these results demonstrate that the top relevant clusters identified by LEGEND comprise genes that are co-functional within context-specific GOBPs and KEGG pathways.

**Figure 5:**
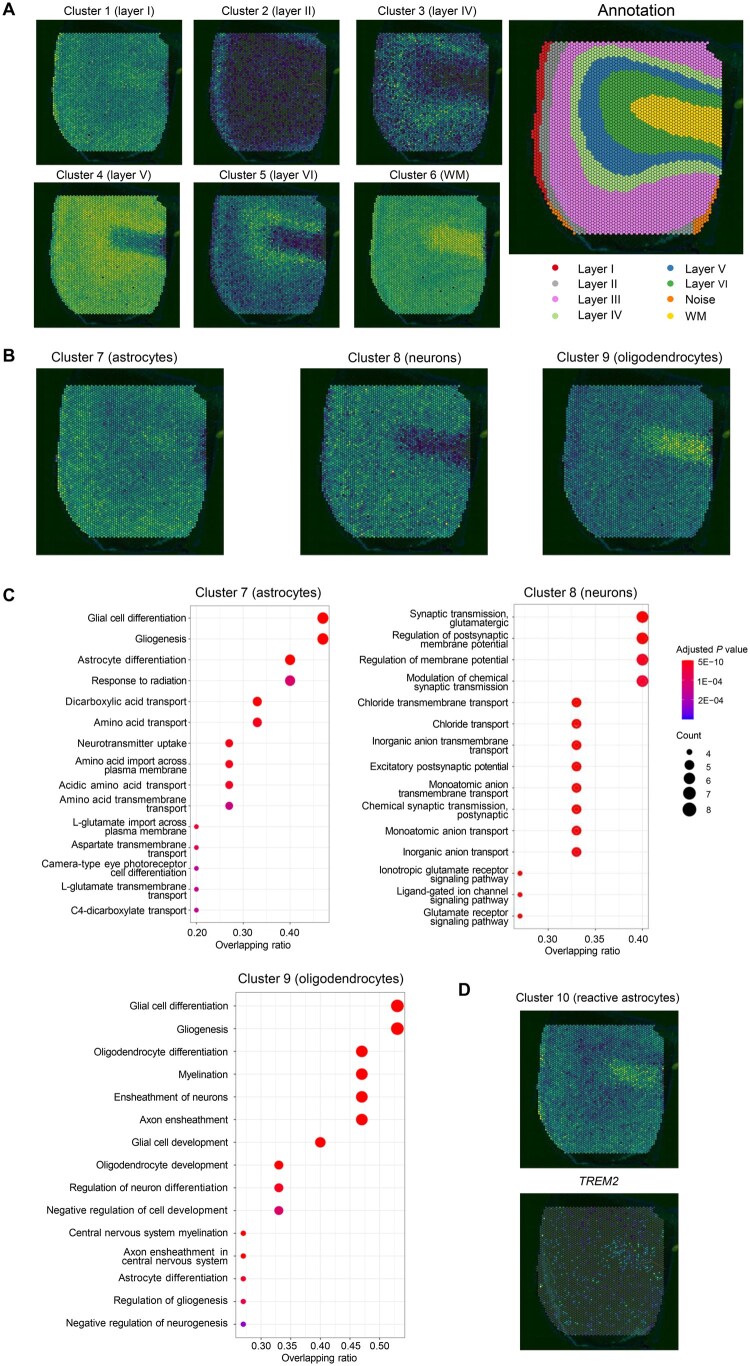
Biological interpretation of LEGEND-identified co-expressed gene clusters in the hMTG-AD-SRT dataset from MTG of AD patients **A**. LEGEND-identified gene clusters coincide with the anatomical structure of cortical layers in human brain. The left six panels show the aggregated spatial expression patterns (module scores) of six gene clusters (Clusters 1–6) identified by LEGEND. The right panel displays the annotated anatomical structure of cortical layers. Clusters 1–6 overlap with the cortical layers I, II, IV, V, VI, and WM, respectively. Brightness in each panel is positively correlated with the aggregated gene expression levels. **B**. LEGEND-identified gene clusters reflect immune cell distributions in cortex layers. The three panels represent the aggregated spatial expression patterns of three LEGEND-identified gene clusters (Clusters 7–9). Genes of Clusters 7, 8, and 9 are primarily expressed in cortical layer I, layers II–VI, and WM, respectively. These patterns reflect the typical distributions of astrocytes, neurons, and oligodendrocytes, respectively. **C**. The most significantly enriched BPs in gene clusters 7–9. The X-axis represents overlapping ratio (the proportion of cluster genes involved in the BP). Circle size indicates the number of genes in the pathway, while red color indicates significantly enriched BPs. **D**. A LEGEND-identified gene cluster (Cluster 10) exhibits significantly elevated transcriptional activity in the WM (left). This pattern mirrors the pathological distribution of reactive astrocytes in AD brain and is recapitulated by the spatial expression of the *TREM2* gene (right), a member gene of Cluster 10 and linked to microglial activation in AD. WM, white matter.

We delved deeper into the biological significance of these gene clusters by examining the overlap between the aggregated expression patterns of their component genes and the anatomical tissue structures or cell type distributions (File S1, see the “Visualization of clusters’ aggregated gene expression patterns” section for details). Six gene clusters identified in the hMTG-AD-SRT dataset exhibited spatial expression patterns closely aligned with the laminar architecture of the human cortex (**Figure 5**A): Clusters 1 to 6 overlapped with cortical layers I, II, IV, V, VI, and white matter (WM), respectively. Moreover, the spatial expression patterns overlapped with the distributions of certain brain-resident immune cell types. For instance, higher proportions of astrocytes, neurons (excitatory and inhibitory), and oligodendrocytes were found in layer I, layers II–VI, and WM, respectively [38], corresponding to the areas where genes of Clusters 7, 8, and 9 were over-expressed (Figure 5B). Closer examination revealed that Cluster 7 included marker genes of astrocytes (*e.g.*, *GFAP*, *ALDH1L1*, and *AQP4*), Cluster 8 contained marker genes of neurons (*e.g.*, *GRIN1, GRIN2A*, and *GABRA1*), and Cluster 9 included marker genes of oligodendrocytes (*e.g.*, *OLIG1, CNP*, and *PDGFRA*). These three clusters were respectively enriched for GOBPs relevant to astrocytes (*e.g.*, gliogenesis and glial cell differentiation), neurons (*e.g.*, glutamatergic synaptic transmission and regulation of postsynaptic potential), and oligodendrocytes (*e.g.*, oligodendrocyte differentiation and myelination) (Figure 5C), further confirming their associations with these cell types. More importantly, certain gene clusters revealed spatial characteristics of AD pathology. The transcriptional activity of genes in Cluster 10, which included reactive astrocyte-related genes (*e.g.*, *VIM* and *S100B*), was significantly elevated in WM (Figure 5D), reflecting the spatial distribution of reactive astrocytes in MTG. This observation is consistent with the finding of increased presence of astrocytes in deeper cortical layers and WM in response to the spread of Aβ plaques, neurofibrillary tangles, and neuroinflammation as AD progresses [39]. Intriguingly, the *TREM2* gene, primarily known for its role in regulating microglial activation and phagocytosis [40], was also identified in this cluster. Considering its co-expression with reactive astrocyte-related genes in the same cluster (Figure 5D), *TREM2* may play a role in regulating reactive astrocytes, a hypothesis also proposed in a recent study [41].

### Identifying AD-associated gene–gene interactions

**Figure 6:**
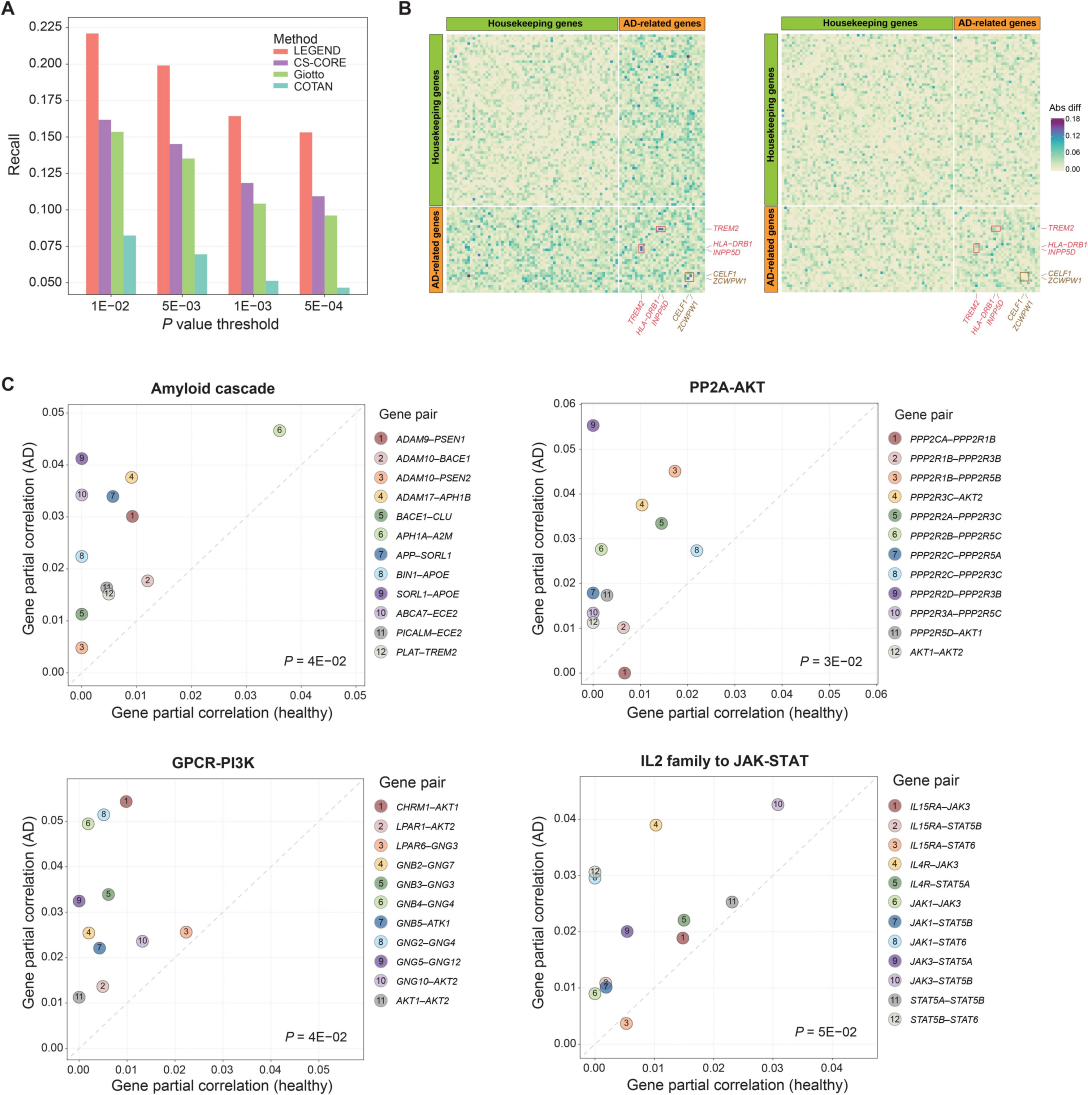
LEGEND reveals disease-associated gene–gene interactions **A**. Prediction of TF–TG interactions. Bar groups represent sets of gene pairs of varying sizes (23.76%, 21.5%, 17.66%, and 16.41%), corresponding to the number of gene pairs with CS-CORE *P* values below the specified thresholds (1E–02, 5E–03, 1E–03, and 5E–04) on the X-axis. Method effectiveness in recovering known TF–TG interactions is quantified as recall scores displayed on the Y-axis. **B**. Heatmaps illustrating the absolute differences in LEGEND-derived partial correlations of 33 AD-associated genes and 66 housekeeping genes between AD and healthy states (left) and between two healthy conditions (right). Darker shades signify greater shifts. Red squares highlight three gene pairs (*TREM2–HLA-DRB1*, *TREM2–INPP5D*, and *CELF1–ZCWPW1*) that show significant correlation shifts in AD but minor changes between healthy conditions. **C**. Scatterplots depicting absolute partial correlations between genes within four established AD-associated gene pathways, including the amyloid cascade, PP2A-AKT, GPCR-PI3K, and JAK-STAT signaling pathways. Spots in each plot represent gene pairs in the signaling pathway. Statistical significance (*P* values from one-sided Student’s *t*-test) for the differences in partial correlations between the AD state and the healthy state is indicated in the lower-right corner of each plot. TF, transcriptional factor; TG, target gene; Abs diff, absolute difference; JAK-STAT, Janus kinase-signal transducer and activator of transcription; GPCR-PI3K, G protein-coupled receptor-phosphatidylinositol 3-kinase; PP2A-AKT, protein phosphatase 2A-protein kinase B.

We evaluated the utility of LEGEND in identifying disease-associated gene–gene interactions by exploring shifts in LEGEND-generated gene co-expression networks from the healthy state to the diseased state. To establish the methodological soundness, we first examined the quality of gene–gene interactions implied by the gene partial correlation matrix derived from the LEGEND-generated gene redundancy matrix (see “Identifying disease-associated gene crosstalk” section). This analysis included a total number of 7102 genes, forming 25,215,651 gene pairs, among which 8239 were curated transcription factor (TF)–target gene (TG) pairs in the Transcriptional Regulatory Relationships Unraveled by Sentence-based Text mining (TRRUST) v2 database [19,42], serving as the ground truth. LEGEND generated the partial correlation matrix for these genes using an scRNA-seq dataset (hPC-healthy-sc) and an SRT dataset (hDLPFC-SRT, slice 151673), both from healthy human prefrontal cortex tissues. Three benchmark methods, including Giotto, CS-CORE, and COTAN, predicted gene–gene interactions using the hPC-healthy-sc dataset alone. Each method assigned “scores” to gene pairs to indicate their likelihood of interaction. For instance, in LEGEND, the score was determined by the partial correlation between gene pairs, while in CS-CORE, it was based on a *P* value derived from a statistical test under the null hypothesis that two genes are uncorrelated. Gene pairs were then ranked by these scores, and the top-ranked (*i.e.*, most likely interacting) gene pairs predicted by each method were selected for further analyses. Specifically, CS-CORE selected four sets of gene pairs of different sizes, containing gene pairs with *P* values below thresholds of 1E–02, 5E–03, 1E–03, and 5E–04, respectively. Similarly, each of the other methods also selected four sets of top-ranked gene pairs, with sizes matching the corresponding sets selected by CS-CORE. Across all four sets, LEGEND consistently outperformed the competing methods, recovering the greatest number of TF–TG interactions, as indicated by its superior recall scores shown in **Figure 6**A, which demonstrates LEGEND’s ability in capturing essential gene relationships.

Subsequently, we compiled a set of 99 genes, consisting of 33 AD-associated genes from previous studies (Table S3) and 66 housekeeping genes [randomly selected from a large pool of 2178 genes cataloged in the Housekeeping and Reference Transcript (HRT) Atlas v1.0 database [43] to form a control gene set]. Three partial correlation matrices of these 99 genes, denoted as $\Phi^{\mathrm{AD}}$, $\Phi^{\mathrm{health}1}$, and $\Phi^{\mathrm{health}2}$, were generated by LEGEND from a pair of AD datasets (hMTG-AD-SRT and hMTG-AD-sc) and two pairs of healthy human MTG datasets (hMTG-health-SRT and hMTG-health-sc; hMTG-health-SRT2 and hMTG-health-sc2). Alterations in gene–gene interactions between two datasets were measured as absolute differences between their partial correlation matrices: $\Delta_{\Phi }^{\mathrm{HD}}:=\;|{\Phi^{\mathrm{health}1}-\Phi }^{\mathrm{AD}}|$ and $\Delta_{\Phi }^{\mathrm{HH}}:=\;|\Phi^{\mathrm{health}1}-\Phi^{\mathrm{health}2}|.$ For easier interpretation, $\Delta_{\Phi }^{\mathrm{HD}}$ and $\Delta_{\Phi }^{\mathrm{HH}}$ are visualized as heatmaps in Figure 6B. For $\Delta_{\Phi }^{\mathrm{HD}}$, we found that gene pairs including at least one AD-associated gene (AD-associated gene pairs) demonstrated significantly greater shifts (Figure 6B, left panel) than those solely comprising housekeeping genes, implying altered gene–gene interactions involving AD-associated genes in the AD context. Furthermore, we employed a paired permutation test to evaluate the statistical significance of partial correlation shifts in $\Delta_{\Phi }^{\mathrm{HD}}$ by comparing them with the non-disease-associated shifts in $\Delta_{\Phi }^{\mathrm{HH}}$. Gene pairs with adjusted *P* values≤0.05 exhibited statistically significant shifts in AD and thus were deemed as disease-associated gene–gene interactions (Figure S7). The methodological details of the test and results are available in the “Statistical test of disease-associated changes in gene–gene interactions” section in File S1. In contrast, $\Delta_{\Phi }^{\mathrm{HH}}$ did not show such disparities (Figure 6B, right panel), aligning with expectations that gene relationships among both AD-related and housekeeping genes remain largely consistent across healthy conditions. For instance, the partial correlations between three gene pairs, including *TREM2–HLA-DRB1*, *TREM2*–*INPP5D*, and *CELF1*–*ZCWPW1*, exhibited the most significant shifts in the AD context but not between the two healthy states, suggesting their interactions are potentially linked to AD. Intriguingly, recent studies have reported that *INPP5D* acts downstream of *TREM2* to regulate the microglial barrier against Aβ toxicity in AD [44]; TREM2 and HLA-DRB gene families are collectively linked to neuroinflammation throughout the AD continuum [45]; and *ZCWPW1* and *CELF1* are co-upregulated in PPARγ-knockdown cell lines associated with AD [46] and in the context of AD risk polymorphisms [47].

We further investigated the altered interactions between genes participating in four established AD-associated signaling pathways, including the amyloid cascade [48], protein phosphatase 2A-protein kinase B (PP2A-AKT) [49], G protein-coupled receptor-phosphatidylinositol 3-kinase (GPCR-PI3K) [49], and Janus kinase-signal transducer and activator of transcription (JAK-STAT) [50] signaling pathways (Table S4), by plotting their partial correlation values in $\Phi^{\mathrm{AD}}$ and $\Phi^{\mathrm{health}1}$ on different axes in scatterplots (Figure 6C). Our analysis revealed that gene pairs within each of the four signaling pathways exhibited statistically significant increases in correlation in the AD dataset compared to the healthy dataset. These observations underscore LEGEND’s ability to detect context-specific alterations in gene–gene interactions, thus facilitating the illumination of molecular mechanisms underlying diseases.

### Detecting genes with designated spatial expression patterns

**Figure 7:**
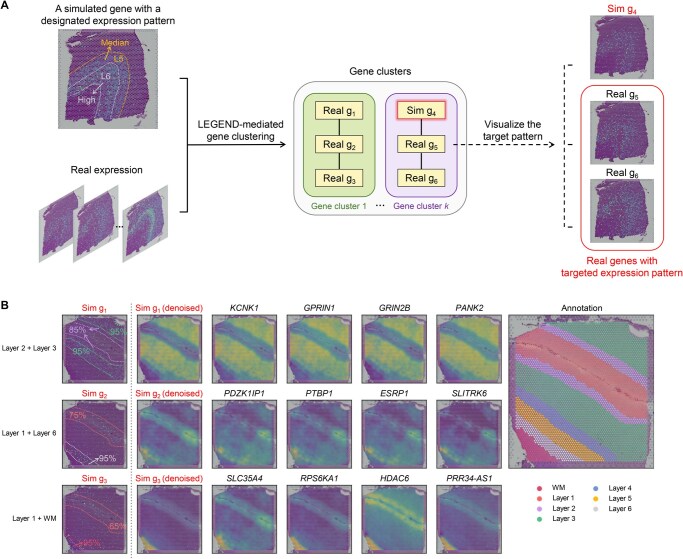
Utilizing LEGEND to pinpoint genes with designated spatial expression patterns **A**. Workflow overview. A simplified example illustrates the strategy to identify genes with high expression in cortex layer 6 (L6) and median expression in cortex layer 5 (L5). A pseudo-gene reflecting this specific expression pattern is simulated and grouped with real genes through LEGEND-mediated gene clustering. Genes clustered with the pseudo-gene are expected to exhibit the targeted expression pattern. **B**. Application to Human DLPFC. The objective is to identify genes in the hDLPFC-SRT dataset that align with three distinct spatial expression patterns: (1) very-high expression in layer 3 and high expression in layer 2; (2) very-high expression in layer 6 and moderate-high expression in layer 1; and (3) very-high expression in WM and moderate expression in layer 1, with low expression in all other layers. The rightmost panel provides ground-truth cortex layer annotations. The leftmost column illustrates the simulated pseudo-genes tailored to the desired patterns, followed by a column showing their denoised expression. The next four columns display the denoised expression patterns of four genes co-clustered with the corresponding pseudo-gene. Sim, simulated.

Identifying genes with designated spatial expression patterns is crucial for uncovering genes associated with biological phenotypes and diseases. For example, certain types of brain-resident immune cells (*e.g.*, reactive astrocytes) in AD exhibit specific distribution patterns across cortical layers during neuroinflammation [39]. Exploring genes that display similar patterns in spatial expression can provide critical clues to molecular mechanisms driving the spatial distribution of these cells. While numerous methods exist for detecting genes differentially expressed in specific regions [51], they are less effective at identifying genes with varying expression levels across multiple regions. To address this limitation, we introduce an innovative approach capable of simulating a pseudo-gene that mirrors the designated spatial expression patterns (File S1, see the “Simulate designated gene spatial expression patterns” section for details). By feeding the pseudo-gene alongside the target SRT dataset to LEGEND, real genes resembling the pseudo-gene in spatial expression patterns can be identified via LEGEND-mediated gene clustering (**Figure 7**A).

To evaluate this approach, we simulated three pseudo-genes for the hDLPFC-SRT (slice 151509) dataset, each with distinct designated expression patterns across the cortex layers of human DLPFC (Figure 7B). The first pseudo-gene is characterized by very-high expression in layer 3 and high expression in layer 2; the second exhibits very-high expression in layer 6 and moderate-high expression in layer 1; the third demonstrates very-high expression in WM and moderate expression in layer 1. Moreover, all three pseudo-genes exhibit low expression in other layers. Gene expression levels are defined as: “very-high” corresponds to the 95th percentile of average expression across all genes, with levels descending through “high” at the 85th percentile, “moderate-high” at the 75th, and “moderate” at the 65th, to “low” at the 35th percentile. Figure 7B showcases that, from LEGEND-generated gene clusters that contain the simulated pseudo-genes, we successfully pinpoint real genes with spatial expression patterns matching the target patterns.

### Improving clustering accuracy of spatial spots and single cells

LEGEND can improve the informational efficiency of feature genes by selecting representative genes with minimized redundancy and maximized relevance from LEGEND-generated gene clusters (see “Selecting an informationally optimal set of feature genes for cell/spot clustering” section). These representative genes are used as inputs for downstream spatial or single-cell clustering algorithms to boost their performance. In this analysis, we employed Leiden and SpaGCN for spatial clustering and Seurat v5 [16] for single-cell clustering. Representative genes were selected from gene clusters generated by LEGEND, LEGEND-no-sc, or scGeneClust from 13 paired SRT and scRNA-seq**/**snRNA-seq datasets (Table 1). For comparative purposes, we also included several competing methods to improve feature efficiency: SpatialDE, Binary Spatial Extraction (BinSpect), and SPARK-eXpedited (SPARK-X) for selecting SVGs in SRT data; and Seurat’s variance stabilizing transformation (VST) and M3Drop (which fits a Michaelis-Menten function to model the relationship between mean expression and dropout rate) for selecting HVGs in scRNA-seq**/**snRNA-seq data. Each method selected a similar number of genes as input to the clustering algorithms.

**Figure 8:**
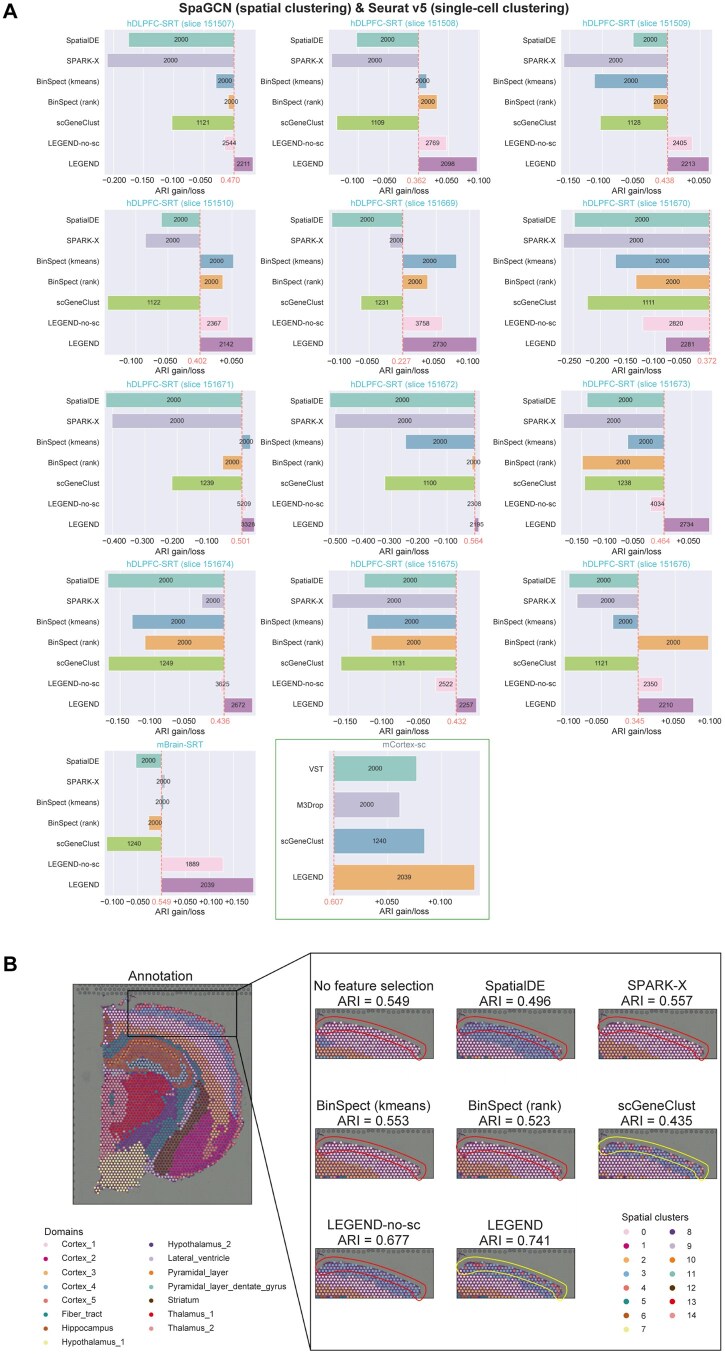
LEGEND improves both single-cell and spatial clustering performance **A**. SpaGCN is employed for spatial clustering across 13 SRT datasets, while Seurat v5 for single-cell clustering in one scRNA-seq dataset (the rectangle-enclosed panel). Both methods utilize feature gene sets selected by LEGEND or six competing methods. The X-axis displays the ARI changes (+, gain; −, loss) compared to baseline performance achieved using the complete gene set (red numbers). The number of genes selected by each method is noted on their bars. **B**. Enhanced tissue domain detection within mouse brain using LEGEND-selected feature gene set. The leftmost panel displays the ground-truth domain labels of the mBrain-SRT dataset, with two adjacent tissue domains, Cortex_4 and Cortex_5, outlined by a black rectangle. The right panels show magnified views of the Cortex_4 and Cortex_5 domains, comparing domain labeling by SpaGCN using feature genes selected by LEGEND and competing methods. Notably, only scGeneClust and LEGEND effectively distinguish Cortex_5 from Cortex_4, as indicated by yellow circles. The accuracy of spatial domain detection is measured using ARI, shown above each right panel. ARI, Adjusted Rand Index; SPARK-X, SPARK-eXpedited; BinSpect, Binary Spatial Extraction; VST, variance stabilizing transformation; M3Drop, fitting Michaelis-Menten function to the relationship between mean expression and dropout-rate.

We first compared the spatial clustering performance using LEGEND-selected genes *versus* SVGs selected by the competing methods. Across all 13 SRT datasets, LEGEND outperformed all competing methods in enhancing the performance of both SpaGCN and Leiden, as indicated by the greatest improvements in ARIs and NMIs (**Figure 8**A, Figures S8–S10). Notably, LEGEND’s superiority over scGeneClust and LEGEND-no-sc can be explained as follows: the latter two methods exclude genes only if they are redundant at the cell type level or the tissue domain level, while LEGEND only excludes genes redundant at both levels, thereby preserving more complementary information between the two levels. A key observation was that Cortex_4 and Cortex_5, two adjacent tissue domains in Figure 8B, were accurately differentiated by SpaGCN only when using feature genes selected by LEGEND and scGeneClust. We posit that genes capable of discriminating between these two domains have relatively stable expression across tissue domains but vary across cell types. Consequently, they were overlooked by SVG selection methods but retained by LEGEND. Similarly, the single-cell clustering performance of LEGEND-selected genes was compared to that of HVGs selected by the competing methods using the mCortex-sc dataset. We found that, compared to the competing methods, LEGEND also exceled in enhancing the performance of Seurat v5 (Figure 8A). These results collectively affirm LEGEND’s ability to select a subset of informationally efficient genes that simultaneously capture spatial and cell type variabilities, making it instrumental in both spatial and single-cell clustering.

## Discussion

scRNA-seq and SRT allow the discovery of gene co-expression patterns across cell types and tissue domains, revealing their co-functionality and interactions in biological systems and diseases. However, existing methods are limited to either scRNA-seq or SRT data, underutilizing the complementary information between the two data types. This leads to suboptimal co-functionality within identified gene co-expression groups. To address this limitation, we propose LEGEND, an innovative approach that models gene relationships inherent in paired scRNA-seq/snRNA-seq and SRT data by considering gene relevance, redundancy, and complementarity. Based on this modeling, a sophisticated gene clustering method is employed to identify context-specific gene co-expression groups and gene–gene interactions.

To systematically evaluate LEGEND’s efficacy in identifying biologically meaningful groups of co-expressed genes, we compared LEGEND with six competing methods across 19 pairs of SRT and scRNA-seq datasets derived from adult mouse brain, human DLPFC, and human breast cancer. LEGEND-identified co-expressed gene groups demonstrate not only superior co-expression and spatial coherence over those identified by the competing methods but also significant context-specific biological relevance. This is evidenced by extensive pathway enrichment analyses, gene co-function analyses, and the congruence of their aggregated spatial expression patterns with known anatomic architecture and cell type distributions. Notably, LEGEND also facilitates the identification of altered gene–gene interactions associated with diseases by examining variations in gene–gene interaction networks derived from healthy *versus* diseased datasets, as demonstrated by our experiments in which LEGEND successfully reveals altered interactions involving AD-associated genes. Furthermore, we demonstrated LEGEND’s utility in downstream analytical tasks, such as pinpointing genes with designated spatial expression patterns and enhancing the informational efficiency of feature gene set for specific algorithms, *e.g.*, those for spatial and single-cell clustering.

To our knowledge, LEGEND is the first method integrating SRT and scRNA-seq/snRNA-seq data for identifying groups of co-expressed and co-functional genes from perspectives of both cell types and spatial tissue domains, alongside a pioneering solution for identifying genes with predefined spatial expression patterns across tissues. LEGEND’s remarkable performance can be attributed to the effective integration of scRNA-seq and SRT data, and a comprehensive quantification of gene informativeness and gene relationships in terms of redundancy, relevance, and complementarity. Nonetheless, there is still room for LEGEND’s future improvement. For example, faster algorithms for calculating gene relevance, redundancy, and complementarity could replace the computationally intensive calculations of MI involving continuous variables. Lastly, LEGEND-identified gene co-expression groups can potentially serve as genomic “contexts” for the derivation of semantically rich distributed gene representations analogous to the learning of word embeddings from textual contexts in linguistic models.

## Code availability

LEGEND is freely available as a Python package on GitHub (https://github.com/ToryDeng/LEGEND). The code has also been submitted to BioCode at the National Genomics Data Center (NGDC), China National Center for Bioinformation (CNCB) (BioCode: BT007611), which is publicly accessible at https://ngdc.cncb.ac.cn/biocode/tool/BT007611.

## CRediT author statement

**Tao Deng:** Methodology, Software, Formal analysis, Writing – original draft, Investigation, Visualization, Validation. **Mengqian Huang:** Formal analysis, Investigation, Visualization. **Kaichen Xu:** Formal analysis, Investigation. **Yan Lu:** Formal analysis, Investigation. **Yucheng Xu:** Formal analysis, Investigation, Writing – original draft, Visualization. **Siyu Chen:** Formal analysis, Investigation. **Nina Xie:** Writing – review & editing. **Qiuyue Tao**: Formal analysis, Investigation. **Hao Wu:** Conceptualization, Writing – review & editing. **Xiaobo Sun:** Conceptualization, Supervision, Methodology, Software, Formal analysis, Investigation, Writing – original draft, Writing – review & editing, Validation. All authors have read and approved the final manuscript.

## Supplementary Material

qzaf056_Supplementary_Data
