# Supplementary material for: LEGEND: Identifying Co-expressed Genes in Multimodal Transcriptomic Sequencing Data
Source: Genomics Proteomics Bioinformatics. 2025 Jul 1;23(4):qzaf056. doi: 10.1093/gpbjnl/qzaf056 (PMC12715406; doi:10.1093/gpbjnl/qzaf056)
Supplement: qzaf056_Supplementary_Data [file qzaf056_supplementary_data.zip › File S1.docx]

**File S1 Supplementary text**

**Pseudo-labelling of cells in unannotated single-cell RNA sequencing / single nucleus RNA sequencing datasets**

Given the accessibility of annotated single-cell RNA sequencing (scRNA-seq) dataset, we assume single-cell labels are available by default. In case of unavailable annotated scRNA-seq data, we propose the following approach that groups together cells that are highly likely to be in the same clusters, the indices of which are used as highly confident (HC) pseudo-labels. The algorithm includes the following steps.

*Step 1*: *Gaussian mixture model-based consensus clustering*

Ten Gaussian mixture models (GMMs) are trained using the first 2 to 11 gene-level principal components (PCs) of the expression matrix to group cells into clusters, each generating the posterior probabilities that cells belong to their respective clusters. The number of gene-level PCs used to train GMMs can be determined by the elbow point on the curve of cumulative explained variance ratio. The number of clusters $\nu$ for the GMM can be determined by existing methods [1–3]. Let $\mathbf{L}^{k}\in\mathbb{N}^{n}$ and $\mathbf{P}^{k}\in\mathbb{R}^{n}$ denote the cluster indices and posterior probabilities of all $n$ cells in the $k^{\text{th}}$ GMM clustering. A binary $n\times n$ co-membership matrix $\mathbf{M}^{k}$ in the $k^{\text{th}}$ GMM clustering can be defined as:

|  | $\begin{matrix} \mathbf{M}_{ij}^{k} = \left\{ \begin{matrix} 1, & \text{if}\mathbf{L}_{i}^{k} = \mathbf{L}_{j}^{k}\wedge\min\left( \mathbf{P}_{i}^{k},\mathbf{P}_{j}^{k} \right) \geq0.95 \\ 0, & \text{otherwise} \end{matrix} \right. \end{matrix}$ | (1) |
| --- | --- | --- |

That is, if we are confident enough that cells $i$ and $j$ belong to the same cluster in the $k^{\text{th}}$ GMM clustering, $\mathbf{M}_{ij}^{k}$ is given a value of 1, otherwise 0. All co-membership matrices generated from the 10 GMM clustering are summed up to form a single matrix $\mathbf{W} = \sum_{k=1}^{10} \mathbf{M}^{k}$, in which $\mathbf{W}_{ij}$ represents the frequency that cells $i$ and $j$ are confidently believed to be of the same type. We then define a threshold $\alpha$ (default value 8) to set the values to 0 for the cell pairs that are not consistently in the same group across all GMMs:

|  | $\begin{matrix} \mathbf{W}_{ij} = \left\{ \begin{matrix} \mathbf{W}_{ij}, & \text{if}\mathbf{W}_{ij} \geq\alpha\\ 0, & \text{otherwise} \end{matrix} \right. \end{matrix}$ | (2) |
| --- | --- | --- |

*Step 2*: *clustering and pseudo-labelling of HC cells*

$\mathbf{W}$ in the above step is used to construct a weighted undirected graph, where graph nodes represent cells and $\mathbf{W}_{ij}$ represents the edge weight between node $i$ and $j$. Leiden algorithm is applied on the graph to cluster cells connected by heavy-weighted edges. The top-$m$ largest-sized clusters consisting of cells that belong to the same type with high confidence are preserved as HC clusters. $m$ is determined as:

|  | $m = \min\left( \nu,\left\vert\{\tilde{C}\left\vert\tilde{C}\in\tilde{C}_{leiden}, \right\vert\tilde{C}\vert\geq n_{1}\} \right\vert\right)$ | (3) |
| --- | --- | --- |

Here $\nu$ represents the estimated number of clusters in GMM clustering, and $\tilde{C}_{leiden}$ represents the set of cell clusters generated by Leiden. $\left| \cdot\right|$ denotes the cardinality of a set and $n_{1}\in\mathbb{N}^{+}$ is a predefined number (default value 10).

**Calculation of mutual information involving continuous variables**

In our study, we leverage a k-nearest neighbor-based algorithm to estimate the mutual information (MI) between two continuous variables (*e.g.*, redundancy) or between a continuous variable and a discrete variable (*e.g.*, relevance) [4,5]. Let $\mathrm{MI}\left( X,Y \right)$ denote the MI between two variables $X$ and $Y$, and $H\left( \cdot\right)$ denote the entropy of a variable, we have:

$$\begin{matrix} MI\left( X,Y \right) & = H\left( X \right) + H\left( Y \right) - H\left( X,Y \right) (4a) \\ & = H\left( Y \right) - H\left( Y | X \right) (4b) \end{matrix}$$

Briefly, the algorithm first leverages the Kozachenko–Leonenko estimator [6] to estimate a probability density $\mu_{i}$ for each observation $x_{i}$. For $x_{i}$, it calculates the distance from its k-th nearest neighbor, denoted by $\delta_{i}$, and then calculates the probability of observing exact $K$ neighbors within $\delta_{i}$ via a trinomial formula to obtain $\mu_{i}$. Then, the entropy $H\left( x_{i} \right)$ within $x_{i}$’s k-nearest neighbor space can be calculated as the expected value of $\ln\mu_{i}$ which is a function of $K$, $N$ (number of observations) and $\delta_{i}$, and $H\left( X \right)\approx\left[ \sum_{i=1}^{N} H\left( x_{i} \right) \right]/N$. $H\left( Y \right)$, $H\left( X,Y \right)$, and $H\left( Y|X \right)$ can be approximated in a similar way. $\mathrm{MI}\left( X,Y \right)$ is calculated according to Equation 4a in case of two continuous variables, or Equation 4b in case of one continuous and one discrete variable.

**Proof of Theorem 1**

When $\mathbf{Red}\left( \boldsymbol{g}_{i},\boldsymbol{g}_{j} \right)<\mathbf{Comp}\left( \boldsymbol{g}_{i},\boldsymbol{g}_{j} \right)$, we have the following transformation and inequality:

|  | $\begin{matrix} \mathbf{Red}\left( \boldsymbol{g}_{i},\boldsymbol{g}_{j} \right)-\mathbf{Comp}\left( \boldsymbol{g}_{i},\boldsymbol{g}_{j} \right) = & \mathrm{MI}\left( \boldsymbol{g}_{i},\boldsymbol{g}_{j} \right)-\mathrm{MI}\left( \boldsymbol{g}_{i},\boldsymbol{g}_{j}\vert\boldsymbol{L} \right) \\ = & H\left( \boldsymbol{g}_{i} \right)-H\left( \boldsymbol{g}_{i}\vert\boldsymbol{g}_{j} \right)-H\left( \boldsymbol{g}_{i}\vert\boldsymbol{L} \right)+H\left( \boldsymbol{g}_{i}\vert\boldsymbol{g}_{j},\boldsymbol{L} \right) \\ = & \mathrm{MI}\left( \boldsymbol{g}_{i}\vert\boldsymbol{L} \right)-\mathrm{MI}\left( \boldsymbol{g}_{i},\boldsymbol{L}\vert\boldsymbol{g}_{j} \right) \\ < & 0 \end{matrix}$ | (5) |
| --- | --- | --- |

**Fine-tuning of gene clusters with Excess of Mass algorithm**

In the maximum spanning tree (MST), two genes connected by an edge with a low relative redundancy index (RRI) value are not sufficiently redundant relative to their complementarity/relevance, making them less likely to be co-functional. Consequently, their edge should be severed to separate them into different gene clusters. The Excess of Mass (EOM) algorithm is leveraged to convert the MST into a hierarchy of gene clusters, represented as a dendrogram, by partitioning the MST at different RRI threshold levels. Specifically, the RRI threshold starts with a low value at the top of the hierarchy, so all genes in the MST belong to a single cluster, which is the root node of the dendrogram. As the RRI threshold increases in a top-down manner, fewer MST edges possess large enough RRI values to be preserved, leading to an increasing number of smaller gene clusters toward the hierarchy’s bottom. In cases where cutting an edge results in two child clusters — one small and the other of a comparable size to their parent cluster — the small child cluster is considered as a noise cluster and thus should be merged back into its parent cluster. We set a minimum cluster size of five for a cluster not to be considered as a noise one. After constructing the cluster tree, MST splits are examined in a top-down manner. An edge split is considered to truly generate new clusters only when both new clusters have at least five genes. This process simplifies the cluster tree, which then only consists of persistent gene clusters as branches that gradually lose their genes as the RRI increases.

To further select “high quality” gene clusters from the hierarchy, we define the stability coefficient for a cluster $i$ within the hierarchy as:

|  | $SC\left( i \right) = \sum_{p\in cluster i} \left( \mathbf{RRI}_{p}-\mathbf{RRI}_{\mathrm{birth}} \right)$ | (6) |
| --- | --- | --- |

Here, $\mathbf{RRI}_{\mathrm{birth}}$ and $\mathbf{RRI}_{p}$ correspond to the RRI threshold level when cluster $i$ splits off from its parent cluster and becomes its own cluster, and the RRI threshold level when gene $p$ falls out of cluster $i$. The EOM algorithm proceeds as follows: starting with the leaf nodes as selected clusters, the algorithm traverses up the cluster tree. For a cluster $i$, if the sum of its children’s stability coefficients is greater than its own, then $SC\left( i \right)$ is updated to be the sum value. Otherwise, cluster $i$ is considered as a sufficiently stable cluster and thus selected, while all its descendant clusters are unselected. The algorithm continues until it reaches the root node, outputting the set of selected clusters as the final result.

**Intra-cluster co-expression quality metric**

To evaluate the spatial expression similarity between genes within the same cluster, we propose an intra-cluster co-expression quality (ICQ) metric based on computer vision techniques. Specifically, we apply the Canny Edge Detector (also known as Canny operator) and Patch Brightness Detector to the normalized spatial expression matrix $\mathbf{X}^{u}\boldsymbol{\in}\mathbb{R}^{N_{x}\times N_{y}}$ of gene $u$, where $N_{x} \mathrm{and} N_{y}$ represent number of spatial spots along the horizontal and vertical directions of the spatial map. Out-of-tissue spatial spots are all padded with 0s in$\mathbf{X}^{u}$.

We first smoothen $\mathbf{X}^{u}$ with a convolutional Gaussian kernel, obtaining a denoised expression matrix ${\tilde{\boldsymbol{X}}}^{u}$. The Canny Edge Detector is then applied on ${\tilde{\boldsymbol{X}}}^{u}$ to generate an edge trait matrix $\mathcal{C}^{u}$, which reflects the expression continuity of gene $u$. We apply the Patch Brightness Detector by segmenting ${\tilde{\boldsymbol{X}}}^{u}$into small patches containing $k\times k$spots, where $k\in\{1, 3, 5\}$, from which three patch brightness mean matrices $\mathcal{A}^{u}\left( k \right)$and two variance matrices $\mathcal{S}^{u}\left( k \right)$(eligible only for $k\boldsymbol{=}3, 5$) are calculated:

|  | $\mathcal{A}_{i,j}^{u}\left( k \right) = \mathrm{avg}\left( \boldsymbol{X}_{s\left( k \right),t\left( k \right)}^{u} \right),\mathcal{S}_{i,j}^{u}\left( k \right) = \mathrm{std}\left( \boldsymbol{X}_{s\left( k \right),t\left( k \right)}^{u} \right),$  $1\leq i\leq N_{x}, 1\leq j\leq N_{y}$ | (7) |
| --- | --- | --- |
|  | $s(k) = \left[ i-\frac{\left( k-1 \right)}{2}:i+\frac{\left( k-1 \right)}{2} \right], t(k) = \left[ j-\frac{\left( k-1 \right)}{2}:j+\frac{\left( k-1 \right)}{2} \right]$ | (8) |

Finally, the ICQ matrix $S^{\mathcal{c}}$ of cluster $\mathcal{c}$is calculated as the average Pearson correlation between its gene pairs’ $\mathcal{C}^{u}$, $\mathcal{A}^{u}\left( k \right)$ and $\mathcal{S}^{u}\left( k \right)$:

|  | $S_{u,v}^{\mathcal{c}} = \left\{ \begin{aligned} & avg\left( \rho_{u,v}\left( \Xi^{u},\Xi^{v} \right) \right);\Xi\in\{\mathcal{A}\left( k \right),\mathcal{S}\left( k \right),\mathcal{C}\}, k\in\{1,3,5\},u\neq v \\ & 0, u=v \end{aligned} \right.$and $\boldsymbol{S}^{\mathcal{c}}\in\mathbb{R}^{d\times d}$ | (9) |
| --- | --- | --- |

Here, $u,v$ represent any two genes within cluster $\mathcal{c}$, while $d$ denotes the number of genes within the cluster $\mathcal{c}$.

**Defining Alzheimer’s disease-related and brain-related Gene Ontology** **biological processes and Kyoto Encyclopedia of Genes and Genomes pathways**

Child terms of “generation of neurons (GO:0048699)”, “neuron death (GO:0070997)”, “neurogenesis (GO:0022008)”, “apoptotic process (GO:0006915)” and “axonogenesis (GO:0007409)” in the Gene Ontology (GO) database are defined as Alzheimer’s disease (AD)-related, given their close relationship to AD pathology. GO biological processes (GOBPs) associated with other neural activities, such as “dendritic spine morphogenesis (GO:0060997)” and their respective child terms, are categorized as brain-related, reflecting general neural-related processes. For Kyoto Encyclopedia of Genes and Genomes (KEGG) pathways, those that are reported to share cellular or molecular mechanisms with the AD (*e.g.*, “hsa00190: oxidative phosphorylation”) are categorized as AD-related, while pathways that have connections with other brain functions are categorized (*e.g.*, “hsa04921: oxytocin signaling pathway”) as brain-related.

**Visualization of clusters’ aggregated gene expression patterns**

To visualize the aggregated gene expression patterns of a cluster on the spatially resolved transcriptomics (SRT) spatial map, we employ the “AddModuleScore” function from the Seurat R package to calculate module scores across all spatial spots for that cluster. Specifically, all genes of the cluster are binned based on their average expression, and an equal number of control genes are randomly selected from each bin. The differences between the average expressions of the set of cluster genes and the set of control genes serve as the cluster’s module scores across all spatial spots. Finally, the clusters’ module scores at every spatial spot are colored and displayed on the SRT spatial map, using a color spectrum that corresponds to the range of the module scores. This approach allows us to better understand how the spatial distribution of gene expression relates to the anatomical tissue structure.

**Statistical test of disease-associated changes in gene–gene interactions**

We employed a paired permutation test to assess the significance of changes in AD-associated gene–gene interactions. Specifically, we calculated health–disease (HD) shifts ($\boldsymbol{\Delta}_{\boldsymbol{\Phi}}^{\mathrm{HD}} := \left| {\boldsymbol{\Phi}^{health1}-\boldsymbol{\Phi}}^{\mathrm{AD}} \right|$), which measure differences in partial correlations between the disease ($\boldsymbol{\Phi}^{\mathrm{AD}}$) and the first health ($\boldsymbol{\Phi}^{health1}$) datasets. To account for non-disease-associated variations, we also computed health–health (HH) shifts as the absolute differences between partial correlations from two healthy datasets ($\boldsymbol{\Delta}_{\boldsymbol{\Phi}}^{\mathrm{HH}}:= \left| \boldsymbol{\Phi}^{health1}-\boldsymbol{\Phi}^{health2} \right|$). If a second healthy dataset is unavailable, we generate one via bootstrap sampling from the first. That is, for each cluster of HC spots and cells, we resample with replacement the same number of spots and single cells to constitute the bootstrap sample.

Subsequently, a paired permutation test was performed using $\boldsymbol{\Delta}_{\boldsymbol{\Phi}}^{\mathrm{HD}}$and $\boldsymbol{\Delta}_{\boldsymbol{\Phi}}^{\mathrm{HH}}$. To establish the null distribution under the null hypothesis that the partial correlation shifts exhibit no difference between the two groups (HD *vs*. HH), shifts from both groups were mixed and randomly assigned to either group A ($\boldsymbol{\Delta}_{\boldsymbol{\Phi}}^{A}$) or B ($\boldsymbol{\Delta}_{\boldsymbol{\Phi}}^{B}$) as follows:

|  | $\left\{ \begin{aligned} \boldsymbol{\Delta}_{\boldsymbol{\Phi}}^{A}\left( i,j \right) :=\boldsymbol{\Delta}_{\boldsymbol{\Phi}}^{\mathrm{HH}}\left( i,j \right) \text{or} \boldsymbol{\Delta}_{\boldsymbol{\Phi}}^{\mathrm{HD}}(i,j) \\ \boldsymbol{\Delta}_{\boldsymbol{\Phi}}^{B}\left( i,j \right) :=\boldsymbol{\Delta}_{\boldsymbol{\Phi}}^{\mathrm{HD}}\left( i,j \right) \text{or }\boldsymbol{\Delta}_{\boldsymbol{\Phi}}^{\mathrm{HH}}(i,j) \end{aligned} \right., \forall i,j\in\left[ 1,G \right],$ | (10) |
| --- | --- | --- |

where $G$ is the total number of genes. The average pairwise difference between the two groups of shifts was calculated as

|  | ${\bar{\boldsymbol{\Delta}}}_{\boldsymbol{\Phi}} = \frac{\sum_{1\leq i<j\leq G} \boldsymbol{\Delta}_{\boldsymbol{\Phi}}^{A}\left( i,j \right)-\boldsymbol{\Delta}_{\boldsymbol{\Phi}}^{B}\left( i,j \right)}{\binom{G}{2}},$ | (11) |
| --- | --- | --- |

where $\binom{G}{2}$ is the binomial coefficient. This process was repeated $T=10,000$ times to obtain the distribution of differences under the null hypothesis ${\bar{\boldsymbol{\Delta}}}_{\Phi}^{1},\cdots,{\bar{\boldsymbol{\Delta}}}_{\boldsymbol{\Phi}}^{T}$. For each gene pair, the one-sided *P* value of its observed shift difference $\boldsymbol{\Delta}_{\boldsymbol{\Phi}}^{\mathrm{diff}}\left( i,j \right)=\boldsymbol{\Delta}_{\boldsymbol{\Phi}}^{\mathrm{HD}}\left( i,j \right)-\boldsymbol{\Delta}_{\boldsymbol{\Phi}}^{\mathrm{HH}}\left( i,j \right)$ was computed as

|  | $p_{i,j} = \frac{\sum_{t=1}^{T} 1_{\left\{ {\bar{\boldsymbol{\Delta}}}_{\boldsymbol{\Phi}}^{t}\geq\boldsymbol{\Delta}_{\boldsymbol{\Phi}}^{\mathrm{diff}}\left( i,j \right) \right\}}}{T}.$ | (12) |
| --- | --- | --- |

*P* values were then adjusted using the Benjamini–Hochberg procedure to control the false discovery rate. Significant gene pairs (adjusted $P\leq0.05$) indicate disease-associated interaction changes.

The adjusted *P* values of housekeeping and AD-associated gene pairs are visualized in the heatmap of Figure S7A. AD-associated gene pairs show a much higher proportion of statistically significant interactions compared to housekeeping gene pairs, as illustrated by the violin plots of adjusted *P* values for the two groups (Figure S7B). Notably, three gene pairs — {*TREM2*, *HLA-DRB1*}, {*TREM2*, *INPP5D*}, and {*CELF1, ZCWPW1*} — exhibit significantly larger HD shifts with adjusted *P* values close to zero. These interaction changes align with previous studies linking these pairs to AD. Detailed biological evidence for these gene pairs is provided in the “Identification of AD-associated gene interactions” subsection in Results.

**Simulate designated gene spatial expression patterns**

We introduce a generalized linear model (GLM)-based method to simulate a pseudo-gene with desired spatial expression patterns, based on which real genes with similar expression patterns can be identified via muLtimodal co-Expressed GENes finDer (LEGEND)-mediated gene clustering. This task is not trivial — on one hand, given no prior knowledge about which real genes exhibit the target expression pattern, existing reference-based simulation methods, as seen in Andersson et al. [7], fail to pinpoint an appropriate gene for reference. On the other hand, without referring to real target data, reference-free simulation methods like Spatially Resolved Transcriptomic simulator (SRTsim) [8] fall short of generating SRT data with consistent data properties.

Our method allows specifying precise gene expression levels in targeted tissue regions. Specifically, let $G$ represent the set of all genes and $S$ the set of all spots in the target dataset. The size-factor-normalized read counts of any gene $i\in G$ at spot $j \in S$, $X_{i,j}$, follow a negative binomial distribution:

|  | $X_{i,j}\sim\mathrm{NB}\left( \mu_{i},s_{i} \right)$ | (13) |
| --- | --- | --- |

where $\mu_{i}$ and $s_{i}$ are mean and dispersion parameters, respectively. Then we have the following equations:

|  | $\sigma_{i}^{2}=\mu_{i}+\frac{\mu_{i}^{2}}{s_{i}}\Longrightarrow\text{cv}_{i}^{2}=\frac{1}{\mu_{i}}+\frac{1}{s_{i}},$ | (14) |
| --- | --- | --- |

where $\sigma_{i}^{2}$ and $\text{cv}_{i}^{2}$ represent variance and squared coefficient of variation (SCV) of gene $i$, respectively. As proved in Brennecke et al. [9], we have:

|  | $E\left[ {\hat{\text{cv}}}_{i}^{2} \right] \approx a_{0}+\frac{a_{1}}{\hat{\mu}_{i}},$ | (15) |
| --- | --- | --- |
|  | $\hat{\mu}_{i} = \frac{\sum_{j} X_{i,j}}{N},$ | (16) |
|  | ${\hat{\text{cv}}}_{i}^{2} = \frac{\sum_{j} \left( X_{i,j}-\hat{\mu}_{i} \right)^{2}/\left( N-1 \right)}{\hat{\mu}_{i}^{2}},$ | (17) |

where ${\hat{\text{cv}}}_{i}^{2}$ and $\hat{\mu}_{i}$ are sample SCV and mean, respectively. $N$ denotes the total number of spots in the SRT dataset. Note that ${\hat{\text{cv}}}_{i}^{2}$ approximately follows a $\chi^{2}$ or gamma distribution. By taking $\frac{1}{\hat{\mu}_{i}}$ as input, we can use a GLM of the gamma family with an identity link function to perform the regression:

|  | $\sum_{i} \log\left( P\left( {\hat{\text{cv}}}_{i}^{2} \vert\alpha,\beta_{i} \right) \right) = \sum_{i} \left( -\alpha log\left( \beta_{i} \right)-\frac{{\hat{\text{cv}}}_{i}^{2}}{\beta_{i}}+\left( \alpha-1 \right)\log\left( {\hat{\text{cv}}}_{i}^{2} \right) \right)+C_{1},$ | (18) |
| --- | --- | --- |
|  | $E\left[ {\hat{\text{cv}}}_{i}^{2} \vert\alpha,\beta_{i} \right] = \alpha\beta_{i} = \eta_{i} = a_{0}+\frac{a_{1}}{\hat{\mu}_{i}},$ | (19) |

where $\alpha=\frac{1}{\sigma}$ is a constant, and $\sigma$ denotes the GLM dispersion parameter. Let the natural parameter of the GLM be $\theta_{i} = \frac{-1}{E\left[ {\hat{\text{cv}}}_{i}^{2} | \alpha,\beta_{i} \right]},$ then we have:

|  | $\sum_{i} \log\left( P\left( {\hat{\text{cv}}}_{i}^{2} \vert\alpha,\beta_{i} \right) \right)\propto\sum_{i} \left( \frac{\theta_{i}{\hat{\text{cv}}}_{i}^{2}-\log\left( -\frac{1}{\theta_{i}} \right)}{\sigma}+\frac{1-\sigma}{\sigma}\log\left( {\hat{\text{cv}}}_{i}^{2} \right) \right),$ | (20) |
| --- | --- | --- |

from which coefficients $a_{0}$ and $a_{1}$ can be estimated using the Fisher scoring method.

Assume that we aim to simulate a gene with a top $t\%$expression level in a spatial region $S_{1}\in S$, and the gene conforms to a negative binomial (NB) distribution with mean $\mu$ and dispersion $s$. We first calculate and rank ascendingly the average expressions of all genes in the target dataset, denoted as $L$. Then, $\mu$ and the corresponding SCV, $\text{cv}$, can be estimated as:

|  | $\mu= L@t\%,$ | (21) |
| --- | --- | --- |
|  | $\text{cv}^{2} \approx E\left[ \text{cv}^{2} \right]= \alpha_{0}+\frac{\alpha_{1}}{\mu}.$ |  |

According to Equation 14, $s$ can be computed as:

|  | $s = \frac{1}{\text{cv}^{2}-\frac{1}{\mu}}.$ | (22) |
| --- | --- | --- |

Let $N_{1}$ denote the number of spots within $S_{1}$ region. For each spot $s_{j}\in S_{1}$, the median gene expression across all genes is calculated, based on which $S_{1}$ is reordered ascendingly as $\tilde{S}_{1}$. Next, we simulate $N_{1}$ observations from $NB\left( \mu,s \right)$, which are sorted in ascending order and assigned to correspondingly ranked spots in $\tilde{S}_{1}$. This ordered assignment helps to preserve the typical spatial gene expression structure inherent in the SRT dataset. Thereby, we obtain a simulated gene expressed at the top $t\%$ quantile level within the $S_{1}$ region. We can repeat this procedure to make the simulated gene to be expressed at specified quantile levels within arbitrary tissue regions.

**Algorithms for spatial and single-cell clustering using selected feature genes**

Here, we compared the feature genes selected by LEGEND and nine benchmark feature selection methods in facilitating spatial and single-cell clustering. These pre-selected feature genes were input into Leiden and SpaGCN for spatial clustering, and Seurat version 5 (Seurat v5) for single-cell clustering. The default parameters were adopted for all clustering and feature selection methods. Note that the built-in feature selection step was disabled in each clustering method so that they use all the pre-selected feature genes. A brief introduction of the clustering methods is provided below.

*Leiden*

We used the function “leiden()” in SCANPY (v1.9.3) for Leiden clustering. This function constructs a k-nearest-neighbor (kNN) graph ($k=15$) of spots based on their gene expression differences measured by Euclidean distance. The graph is then iteratively partitioned into spot clusters, optimizing for modularity and cluster connectivity.

*SpaGCN*

We employed SpaGCN (v1.2.7) to initially build a graph, with nodes representing spots, and edge weights indicating the proximity and histological similarity of connected spots. Spot embeddings are then generated using a graph convolutional network, on which a clustering is performed to group spots into tissue domains.

*Seurat*

Seurat (v5.0.1) begins by constructing a kNN graph of cells from the Euclidean distances between their expression levels. Subsequently, a shared nearest neighbor (SNN) graph is created by measuring the neighborhood overlap using the Jaccard index between cell pairs. Finally, the Leiden algorithm is applied to partition the SNN graph into distinct cell clusters.

**Impact of the number of selected feature genes on single-cell/spatial clustering performance**

LEGEND selects $100\times\omega\% (0<\omega<1)$ top relevant genes from each generated gene module to serve as input to downstream clustering algorithms. To evaluate the impact of the number of selected feature genes on clustering performance, we tested different $\omega$ values (10%, 20%, 40%, and 60%). As shown in Figures S2 & S3, Adjusted Rand Index (ARI) and Normalized Mutual Information (NMI) metrics do not improve for most datasets when $\omega$ exceeds 20%, suggesting that lower-ranked relevant genes provide little extra information for both spatial spot and single-cell clustering conditional on the top 20% relevant genes. Therefore, selecting genes beyond the top 20% relevant ones does not significantly improve the signal-to-noise ratio of the data but incurs unnecessary computational costs. In a nutshell, $\omega=20\%$ is a reasonable default setting for determining the number of selected feature genes in LEGEND.

**References**

[1] Traag VA, Waltman L, van Eck NJ. From Louvain to Leiden: guaranteeing well-connected communities. Sci Rep 2019;9:5233.

[2] Melchor SJ, Ewald SE. Disease tolerance in *Toxoplasma* infection. Front Cell Infect Microbiol 2019;9:185.

[3] Sun X, Gao J, Jin P, Eng C, Burchard EG, Beaty TH, et al. Optimized distributed systems achieve significant performance improvement on sorted merging of massive VCF files. Gigascience 2018;7:giy052.

[4] Kraskov A, Stögbauer H, Grassberger P. Estimating mutual information. Phys Rev E Stat Nonlin Soft Matter Phys 2004;69:066138.

[5] Ross BC. Mutual information between discrete and continuous data sets. PLoS One 2014;9:e87357.

[6] Kozachenko LF, Leonenko NN. Sample estimate of the entropy of a random vector. Problemy Peredachi Inf 1987;23:9–16.

[7] Andersson A, Bergenstråhle J, Asp M, Bergenstråhle L, Jurek A, Fernández Navarro J, et al. Single-cell and spatial transcriptomics enables probabilistic inference of cell type topography. Commun Biol 2020;3:565.

[8] Zhu J, Shang L, Zhou X. SRTsim: spatial pattern preserving simulations for spatially resolved transcriptomics. Genome Biol 2023;24:39.

[9] Brennecke P, Anders S, Kim J K, Kołodziejczyk AA, Zhang X, Proserpio V, et al. Accounting for technical noise in single-cell RNA-seq experiments. Nat Methods 2013;10:1093–5.
