# Supplementary material for: LEGEND: Identifying Co-expressed Genes in Multimodal Transcriptomic Sequencing Data
Source: Genomics Proteomics Bioinformatics. 2025 Jul 1;23(4):qzaf056. doi: 10.1093/gpbjnl/qzaf056 (PMC12715406; doi:10.1093/gpbjnl/qzaf056)
Supplement: qzaf056_Supplementary_Data [file qzaf056_supplementary_data.zip › supplementary material captions.docx]

**Supplementary material**

**File S1 Supplementary text**

**Figure S1 LEGEND consistently identifies sufficient HC spots with prevalent domain detection methods**

Three domain detection methods, SpaGCN, Bayesian Analytics for Spatial Segmentation (BASS), and GraphST, cluster the spots in the hDLPFC-SRT (slice 151673) dataset into spatial domains (the upper right panel), respectively. LEGEND identifies HC spots within spatial domains detected by each method (the lower right panel). ARI values are calculated between cluster labels of all spots/HC spots and their ground truth annotations (the left panel).

**Figure S2 Impact of the number of selected feature genes on cell/spot clustering performance**

SpaGCN is employed for spatial clustering across 13 SRT datasets, while Seurat v5 for single-cell clustering in an scRNA-seq dataset (the rectangle-enclosed panel). Different percentages of top relevant genes in each LEGEND-generated module are selected as feature genes for both methods, as indicated by the X-axis. LEGEND’s default percentage is 20% (the pink label). The ARI for each percentage is annotated beside each filled circle. Seurat v5, Seurat version 5.

**Figure S3 This figure mirrors Figure S2 except that it shows NMI scores instead**

NMI, Normalized Mutual Information.

**Figure S4 Spatial expression patterns of LEGEND-identified gene clusters in mouse brain (FFPE Visium and Visium HD) and BC samples**

**A.** Gene clusters identified by LEGEND from the mBrain-FFPE dataset are divided into groups of high, medium, and low co-expression quality based on their ICQ metrics. Rows represent a gene cluster randomly selected from each of these groups, respectively. The denoised spatial expression patterns of four genes randomly chosen from the cluster are visualized in each row. **B.** Three gene clusters are each randomly selected from ICQ-based co-expression quality groups (high, medium, and low) of gene clusters identified by LEGEND from the mBrain-HD dataset. **C.** Three gene clusters are each randomly selected from ICQ-based co-expression quality groups (high, medium, and low) of gene clusters identified by LEGEND from the hBC-SRT dataset. FFPE, Formalin-Fixed Paraffin-Embedded; HD, high definition; BC, breast cancer.

**Figure S5 Performance comparison between LEGEND and eight competing gene clustering methods in identifying gene co-expression groups in scRNA-seq and SRT datasets sampled from mouse brain (FFPE Visium and Visium HD) and BC tissues**

**A**. LEGEND is compared with eight competing gene clustering methods in identifying gene co-expression groups in the pair of mBrain-FFPE and mCortex-sc datasets. **B**. The same comparison as in Figure S5A, using the pair of mBrain-HD and mCortex-sc datasets. **C**. The same comparison as in Figure S5A, using the pair of hBC-SRT and hBC-sc datasets.

**Figure S6 KEGG pathway enrichment and co-function analyses of LEGEND-identified gene clusters**

Top relevant gene clusters are collected from datasets of MTG in both healthy and AD individuals, forming a normal group and a disease group. A control group comprising a comparable number of randomly selected genes serves as a benchmark. **A.** The 20 most significantly enriched pathways for each gene group. The X-axis represents the negative logarithm of adjusted *P* values of enrichment significance, while the Y-axis represents the AUC scores of the 20 pathways from gene co-function analysis, where a higher score indicates stronger co-functionality within the pathway. Red color indicates AD-related (also brain-related) pathways, green color indicates only brain-related pathways, and blue color indicates other pathways. **B.** Enrichment maps of the 20 most significantly enriched pathways for each gene group. The connectivity of the network indicates functional associations among pathways. The node color represents pathway’s enrichment significance (negative logarithm of adjusted *P* value), with red color indicative of significantly enriched pathway. Node size denotes the number of genes involved in the pathway. KEGG, Kyoto Encyclopedia of Genes and Genomes.

**Figure S7 Statistical significance of AD-associated gene interactions**

**A**. The heatmap illustrates adjusted *P* values from permutation tests using health–-disease and health–-health shifts. *P* values are not calculated for pairs of identical genes, as indicated by the grey diagonal. Significant gene pairs (adjusted $P\leq0.05$) are colored in red . Notably marked with green and blue squares are three gene pairs ({*TREM2*, *HLA-DRB1*}, {*TREM2*, *INPP5D*}, and {*CELF1*, *ZCWPW1*}) showing adjusted *P* values close to zero. **B.** Violin plots of adjusted *P* values of housekeeping gene pairs (colored in orange) and AD-associated gene pairs (colored in green).

**Figure S8 This figure mirrors Figure 8A except that it shows NMI scores instead**

**Figure S9 LEGEND improves clustering performance in SRT**

Leiden is employed for spatial clustering across 13 SRT datasets, utilizing feature gene sets selected by LEGEND or six competing methods. The X-axis displays the ARI changes (+ gain, − loss) compared to baseline performances achieved using the complete gene set (red numbers). The number of genes selected by each method is noted on their bars.

**Figure S10 This figure mirrors Figure S6 except that it shows NMI scores instead**

**Table S1 List of the 20 most significant enriched KEGG pathways in disease, normal, and control groups**

**Table S2 List of the 20 most significantly enriched GOBPs in disease, normal, and control groups**

**Table S3 List of AD-associated genes**

**Table S4 List of AD-associated gene pathways**
