# Supplementary material for: LEGEND: Identifying Co-expressed Genes in Multimodal Transcriptomic Sequencing Data
Source: Genomics Proteomics Bioinformatics. 2025 Jul 1;23(4):qzaf056. doi: 10.1093/gpbjnl/qzaf056 (PMC12715406; doi:10.1093/gpbjnl/qzaf056)
Supplement: qzaf056_Supplementary_Data [file qzaf056_supplementary_data.zip › Table S2.docx]

**Table S2** **List of the 20 most significantly enriched GOBPs in disease, normal, and control groups**

| **Group** | **GOBP ID** | **GOBP name** | **Relevance to**  **AD/brain** |
| --- | --- | --- | --- |
| **Disease**  **group** | GO:0099003 | Vesicle-mediated transport in synapse | Brain related |
|  | GO:0014003 | Oligodendrocyte development | AD and brain related |
|  | GO:0010001 | Glial cell differentiation | AD and brain related |
|  | GO:0036465 | Synaptic vesicle recycling | Brain related |
|  | GO:0042063 | Gliogenesis | AD and brain related |
|  | GO:0099504 | Synaptic vesicle cycle | Brain related |
|  | GO:0021782 | Glial cell development | AD and brain related |
|  | GO:0061564 | Axon development | AD and brain related |
|  | GO:0007272 | Ensheathment of neurons | AD and brain related |
|  | GO:0010563 | Negative regulation of phosphorus metabolic process | AD and brain related |
|  | GO:0008366 | Axon ensheathment | AD and brain related |
|  | GO:0045936 | Negative regulation of phosphate metabolic process | AD and brain related |
|  | GO:0017156 | Calcium-ion regulated exocytosis | AD and brain related |
|  | GO:0042552 | Myelination | AD and brain related |
|  | GO:2000649 | Regulation of sodium ion transmembrane transporter activity | Others |
|  | GO:0048709 | Oligodendrocyte differentiation | AD and brain related |
|  | GO:0051235 | Maintenance of location | Others |
|  | GO:0051962 | Positive regulation of nervous system development | AD and brain related |
|  | GO:0051960 | Regulation of nervous system development | AD and brain related |
|  | GO:0022010 | Central nervous system myelination | AD and brain related |
| **Normal**  **group** | GO:1901028 | Regulation of mitochondrial outer membrane permeabilization involved in apoptotic signaling pathway | Others |
|  | GO:2001233 | Regulation of apoptotic signaling pathway | AD and brain related |
|  | GO:0033033 | Negative regulation of myeloid cell apoptotic process | Brain related |
|  | GO:2001242 | Regulation of intrinsic apoptotic signaling pathway | AD and brain related |
|  | GO:0097193 | Intrinsic apoptotic signaling pathway | AD and brain related |
|  | GO:0022904 | Respiratory electron transport chain | Others |
|  | GO:0046034 | ATP metabolic process | Others |
|  | GO:2001235 | Positive regulation of apoptotic signaling pathway | AD and brain related |
|  | GO:2000191 | Regulation of fatty acid transport | Brain related |
|  | GO:0061001 | Regulation of dendritic spine morphogenesis | Brain related |
|  | GO:0032890 | Regulation of organic acid transport | Brain related |
|  | GO:0033032 | Regulation of myeloid cell apoptotic process | Brain related |
|  | GO:0099173 | Postsynapse organization | Brain related |
|  | GO:0060997 | Dendritic spine morphogenesis | Brain related |
|  | GO:2000107 | Negative regulation of leukocyte apoptotic process | Brain related |
|  | GO:0050808 | Synapse organization | Brain related |
|  | GO:0033028 | Myeloid cell apoptotic process | Brain related |
|  | GO:0006417 | Regulation of translation | Brain related |
|  | GO:0009988 | Cell-cell recognition | Brain related |
|  | GO:0032368 | Regulation of lipid transport | Brain related |
| **Control**  **group** | GO:0052547 | Regulation of peptidase activity | AD and brain related |
|  | GO:0052548 | Regulation of endopeptidase activity | AD and brain related |
|  | GO:0010951 | Negative regulation of endopeptidase activity | AD and brain related |
|  | GO:0072091 | Regulation of stem cell proliferation | Brain related |
|  | GO:0002604 | Regulation of dendritic cell antigen processing and presentation | Others |
|  | GO:2000009 | Negative regulation of protein localization to cell surface | Others |
|  | GO:0002468 | Dendritic cell antigen processing and presentation | Others |
|  | GO:0051044 | Positive regulation of membrane protein ectodomain proteolysis | Others |
|  | GO:0042255 | Ribosome assembly | Others |
|  | GO:0002577 | Regulation of antigen processing and presentation | Others |
|  | GO:0034755 | Iron ion transmembrane transport | Others |
|  | GO:0051043 | Regulation of membrane protein ectodomain proteolysis | Others |
|  | GO:0048714 | Positive regulation of oligodendrocyte differentiation | Others |
|  | GO:0002181 | Cytoplasmic translation | Others |
|  | GO:0061436 | Establishment of skin barrier | Others |
|  | GO:0014910 | Regulation of smooth muscle cell migration | Others |
|  | GO:0033561 | Regulation of water loss via skin | Others |
|  | GO:1903955 | Positive regulation of protein targeting to mitochondrion | Others |
|  | GO:0014909 | Smooth muscle cell migration | Others |
|  | GO:0045907 | Positive regulation of vasoconstriction | Others |

*Note*: GOBPs, GO biological processes; GO, Gene Ontology; ATP, adenosine triphosphate.
