# Supplementary material for: LEGEND: Identifying Co-expressed Genes in Multimodal Transcriptomic Sequencing Data
Source: Genomics Proteomics Bioinformatics. 2025 Jul 1;23(4):qzaf056. doi: 10.1093/gpbjnl/qzaf056 (PMC12715406; doi:10.1093/gpbjnl/qzaf056)
Supplement: qzaf056_Supplementary_Data [file qzaf056_supplementary_data.zip › Table S3.docx]

**Table S3 List of AD-associated genes**

| **AD-associated genes** | **Reference** |
| --- | --- |
| *APP* | [1] |
| *PSEN1*, *PSEN2* | [2] |
| *TREM2* | [3] |
| *SORL1* | [4] |
| *ABCA7* | [5] |
| *ATXN1* | [6] |
| *CHRNA4* | [7] |
| *MAPT* | [8] |
| *CST3* | [9] |
| *TF* | [10] |
| *GAB2* | [11] |
| *APOE*, *BCR*, *CTSS* | [12] |
| *PLD3*, *UNC5C*, *ADAM10*, *CLU*, *BIN1*, *CD2AP*, *PICALM*, *HLA-DRB1*, *INPP5D*, *MEF2C*, *PTK2B*, *ZCWPW1*, *CELF1*, *FERMT2*, *SLC24A4*, *RIN3* | [13] |
| *IQCK*, *WWOX* | [14] |

**References**

[1]Levy E, Carman MD, Fernandez-Madrid IJ, Power MD, Lieberburg I, Van Duinen SG, et al. Mutation of the Alzheimer’s disease amyloid gene in hereditary cerebral hemorrhage, dutch type. Science 1990;248:1124–6.

[2]Levy-Lahad E, Wasco W, Poorkaj P, Romano DM, Oshima J, Pettingell WH, et al. Candidate gene for the chromosome 1 familial Alzheimer’s disease locus. Sci 1995;269:973–7.

[3]Neumann H, Daly MJ. Variant *TREM2* as risk factor for Alzheimer’s disease. N Engl J Med 2013;368:182–4.

[4]Pottier C, Hannequin D, Coutant S, Rovelet-Lecrux A, Wallon D, Rousseau S, et al. High frequency of potentially pathogenic *SORL1* mutations in autosomal dominant early-onset Alzheimer disease. Mol Psychiatry 2012;17:875–9.

[5]Lacour M, Quenez O, Rovelet-Lecrux A, Salomon B, Rousseau S, Richard AC, et al. Causative mutations and genetic risk factors in sporadic early onset Alzheimer’s disease before 51 years. J Alzheimers Dis 2019;71:227–43.

[6]Zhang C, Browne A, Child D, Divito JR, Stevenson JA, Tanzi RE. Loss of function of *ATXN1* increases amyloid β-protein levels by potentiating β-secretase processing of β-amyloid precursor protein. J Biol Cheistry 2010;285:8515–26.

[7]Cook LJ, Ho LW, Taylor AE, Brayne C, Evans JG, Xuereb J, et al. Candidate gene association studies of the α4 (*CHRNA4*) and β2 (*CHRNB2*) neuronal nicotinic acetylcholine receptor subunit genes in Alzheimer’s disease. Neurosci Lett 2004;358:142–6.

[8]Myers AJ, Pittman AM, Zhao AS, Rohrer K, Kaleem M, Marlowe L, et al. The *MAPT H1c* risk haplotype is associated with increased expression of tau and especially of 4 repeat containing transcripts. Neurobiol Dis 2007;25:561–70.

[9]Bertram L, Tanzi RE. Thirty years of Alzheimer’s disease genetics: the implications of systematic meta-analyses. Nat Rev Neurosci 2008;9:768–78.

[10] Robson KJ, Lehmann DJ, Wimhurst VL, Livesey KJ, Combrinck M, Merryweather-Clarke AT, et al. Synergy between the C2 allele of transferrin and the C282Y allele of the haemochromatosis gene (*HFE*) as risk factors for developing Alzheimer’s disease. J Med Genet 2004;41:261–5.

[11] Reiman EM, Webster JA, Myers AJ, Hardy J, Dunckley T, Zismann VL, et al. *GAB2* alleles modify Alzheimer’s risk in *APOE* ɛ4 carriers. Neuron 2007;54:713–20.

[12] Grupe A, Abraham R, Li Y, Rowland C, Hollingworth P, Morgan A, et al. Evidence for novel susceptibility genes for late-onset Alzheimer’s disease from a genome-wide association study of putative functional variants. Hum Mol Genet 2007;16:865–73.

[13] Giri M, Lü Y, Zhang M. Genes associated with Alzheimer’s disease: an overview and current status. Clin Interv Aging 2016;11:665–81.

[14] Kunkle BW, Grenier-Boley B, Sims R, Bis JC, Damotte V, Naj AC, et al. Genetic meta-analysis of diagnosed Alzheimer’s disease identifies new risk loci and implicates Aβ, tau, immunity and lipid processing. Nat Genet 2019;51:414–30.
