# Supplementary material for: LEGEND: Identifying Co-expressed Genes in Multimodal Transcriptomic Sequencing Data
Source: Genomics Proteomics Bioinformatics. 2025 Jul 1;23(4):qzaf056. doi: 10.1093/gpbjnl/qzaf056 (PMC12715406; doi:10.1093/gpbjnl/qzaf056)
Supplement: qzaf056_Supplementary_Data [file qzaf056_supplementary_data.zip › Table S4.docx]

**Table S4 List of AD-associated gene pathways**

| **Pathway** | **Genes involved in the pathway** | **Reference** |
| --- | --- | --- |
| The amyloid cascade signaling pathway | *ADAM9*, *ADAM10*, *ADAM17*, *BACE1*, *PSEN1*, *PSEN2*, *APH1A*, *APH1B*, *APP*, *BIN1*, *SORL1*, *APOE*, *ABCA7*, *CLU*, *PICALM*, *A2M*, *ECE2*, *PLAT*, *TREM2* | [1] |
| PP2A-AKT signaling pathway | *PPP2CA*, *PPP2R1B*, *PPP2R2A*, *PPP2R3C*, *PPP2R2B*, *PPP2R2C*, *PPP2R2D*, *PPP2R3A*, *PPP2R3B*, *PPP2R5A*, *PPP2R5B*, *PPP2R5C*, *PPP2R5D*, *AKT1*, *AKT2* | [2] |
| GPCR-PI3K signaling pathway | *CHRM1*, *LPAR1*, *LPAR6*, *GNB2*, *GNB3*, *GNB4*, *GNB5*, *GNG2*, *GNG3*, *GNG4*, *GNG5*, *GNG7*, *GNG10*, *GNG12*, *AKT1*, *AKT2* | [2] |
| IL-2 family to JAK-STAT signaling pathway | *IL15RA*, *IL4R*, *JAK1*, *JAK3*, *STAT5A*, *STAT5B*, *STAT6* | [3] |

*Note*: PP2A-AKT, protein phosphatase 2A-protein kinase B; GPCR-PI3K, G protein coupled receptor-Phosphatidylinositol 3-kinases; IL-2, interleukin-2; JAK-STAT, Janus kinase-signal transducer and activator of transcription proteins.

**References**

[1] Gadhave K, Gehi BR, Kumar P, Xue B, Uversky VN, Giri R. The dark side of Alzheimer’s disease: unstructured biology of proteins from the amyloid cascade signaling pathway. Cell Mol Life Sci 2020;77:4163–208.

[2] Long HZ, Cheng Y, Zhou ZW, Luo HY, Wen DD, Gao LC. PI3K/AKT signal pathway: a target of natural products in the prevention and treatment of Alzheimer’s disease and Parkinson’s disease. Front Pharmacol 2021;12:648636.

[3] Rusek M, Smith J, El-Khatib K, Aikins K, Czuczwar SJ, Pluta R. The role of the JAK/STAT signaling pathway in the pathogenesis of Alzheimer’s disease: new potential treatment target. Int J Mol Sci 2023;24:864.
