# Supplementary figures and images for: LEGEND: Identifying Co-expressed Genes in Multimodal Transcriptomic Sequencing Data
Source: Genomics Proteomics Bioinformatics. 2025 Jul 1;23(4):qzaf056. doi: 10.1093/gpbjnl/qzaf056 (PMC12715406; doi:10.1093/gpbjnl/qzaf056)

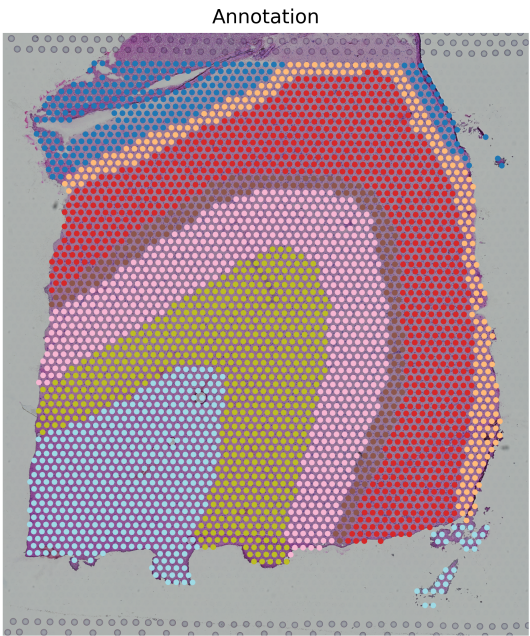

- Layer1
- Layer2
- Layer3
- Layer4
- Layer5
- Layer6
- WM

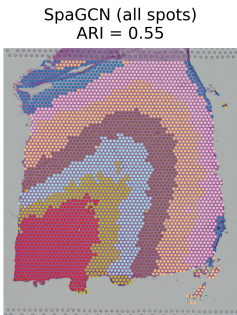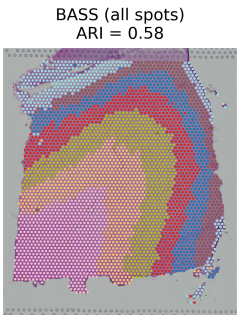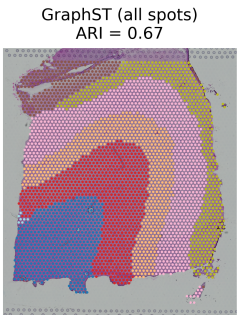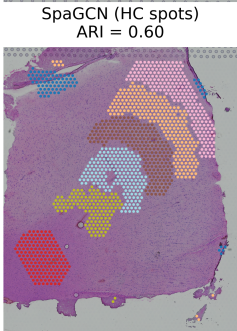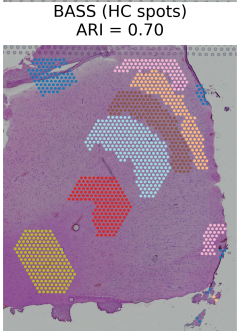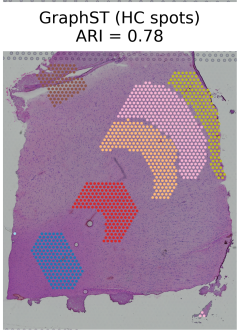

Supplement: qzaf056_Supplementary_Data [file qzaf056_supplementary_data.zip › Figure_S1.pdf]

Leiden (spatial clustering)

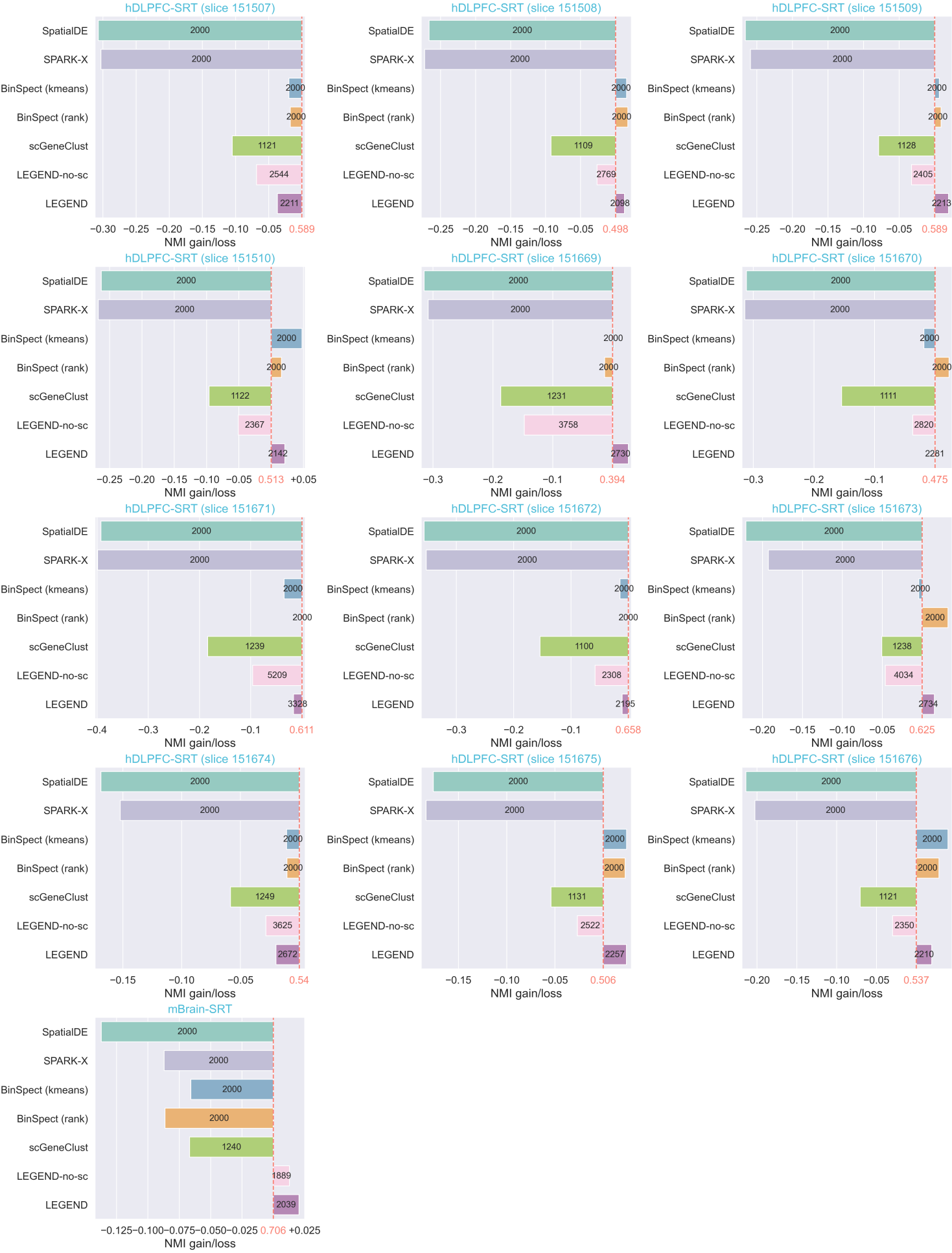

Supplement: qzaf056_Supplementary_Data [file qzaf056_supplementary_data.zip › Figure_S10.pdf]

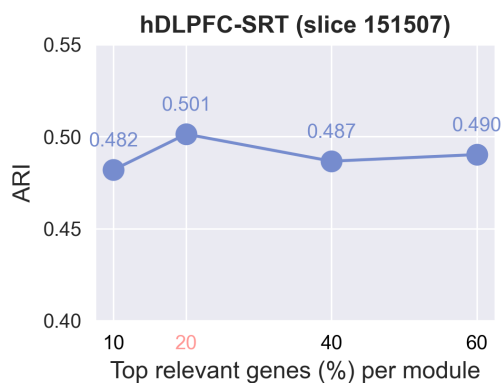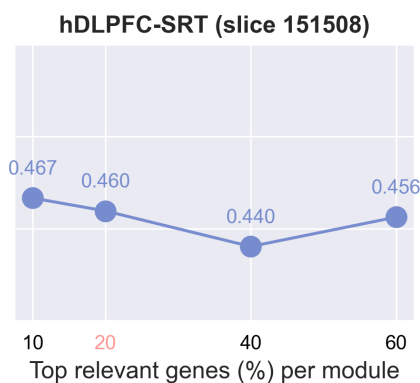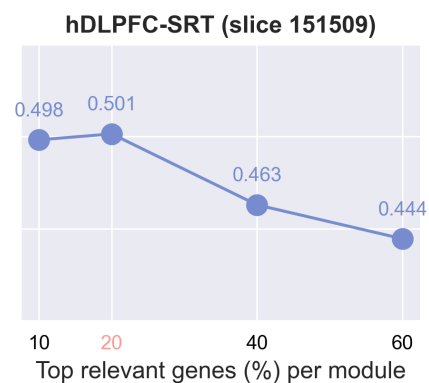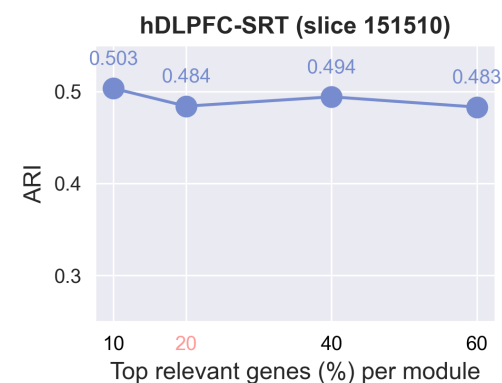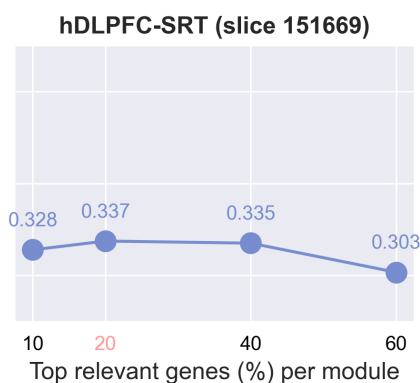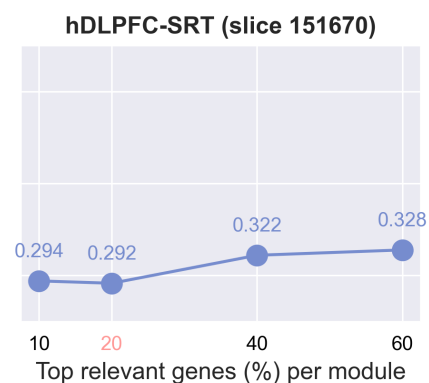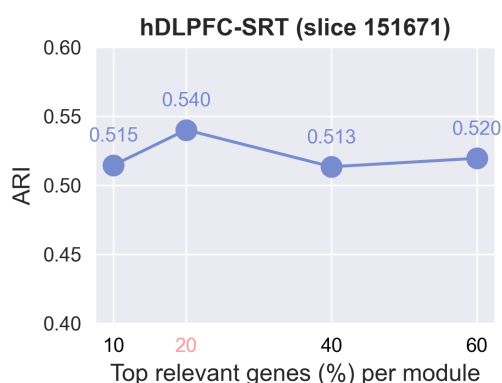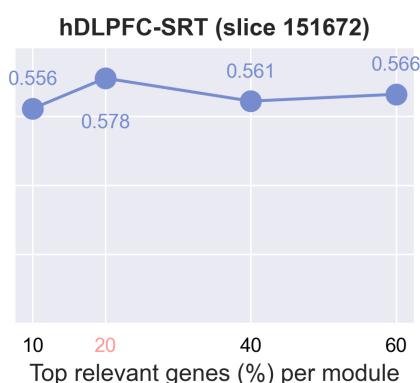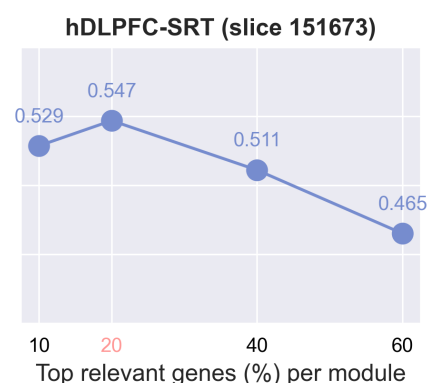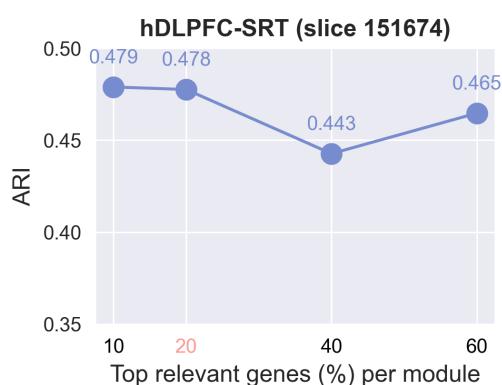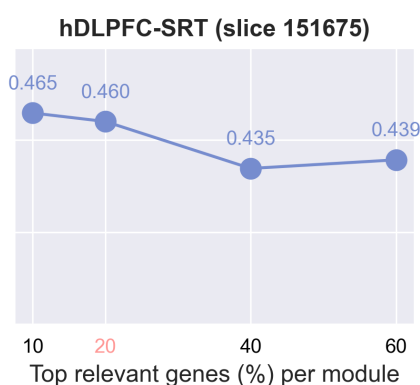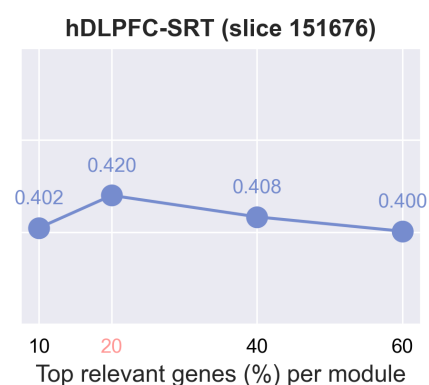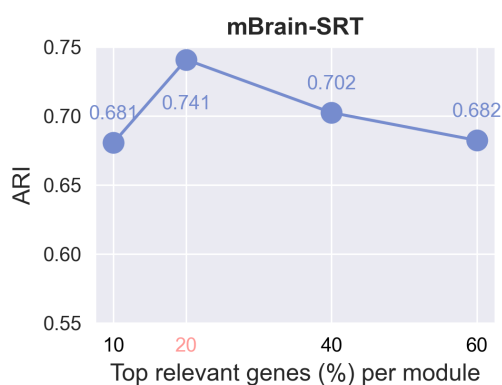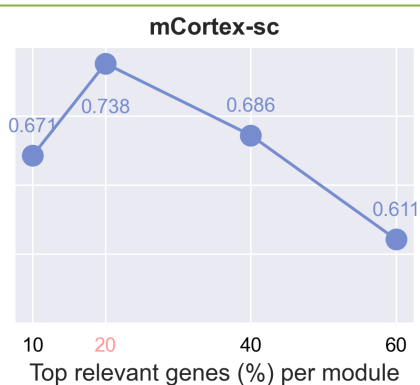

Supplement: qzaf056_Supplementary_Data [file qzaf056_supplementary_data.zip › Figure_S2.pdf]

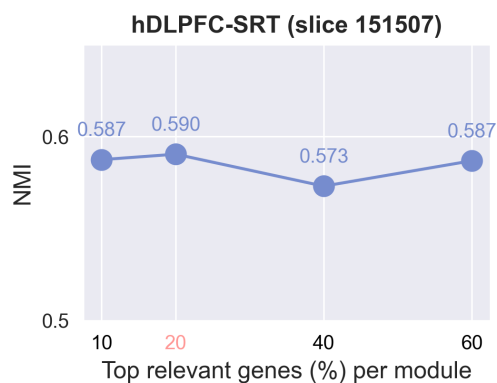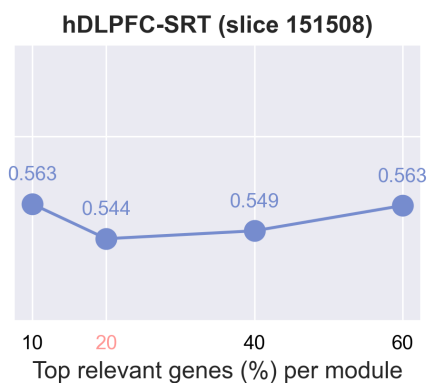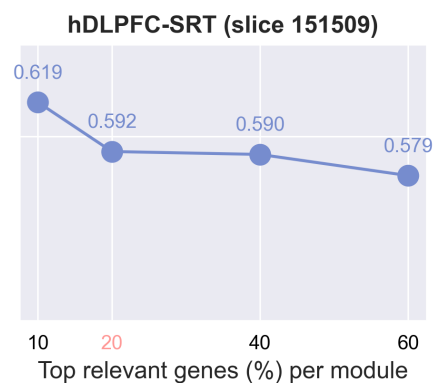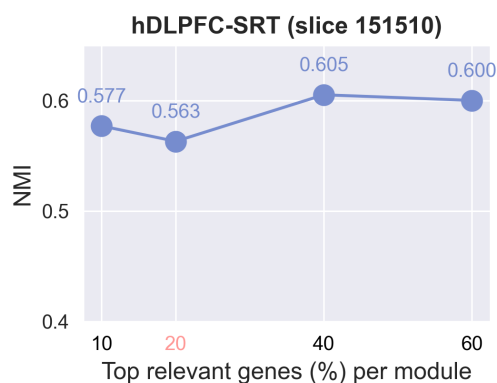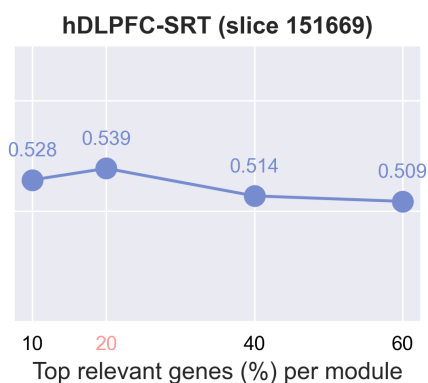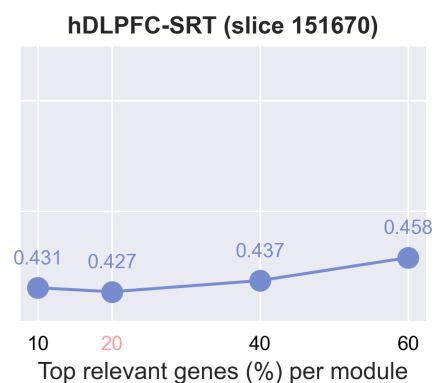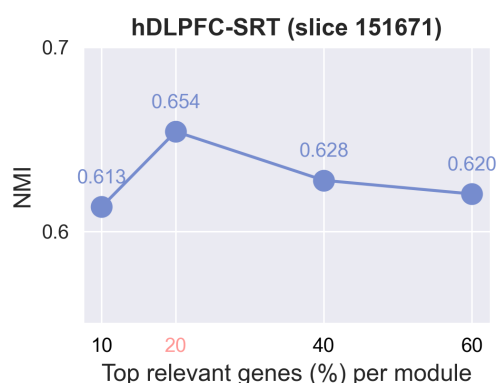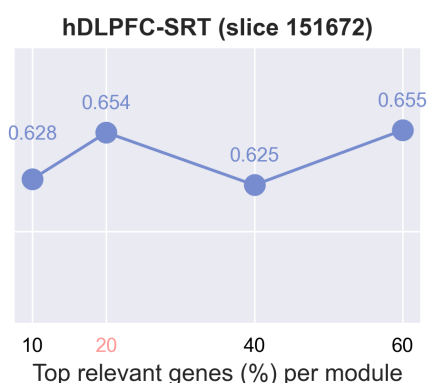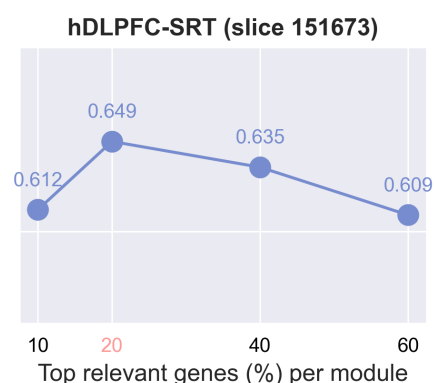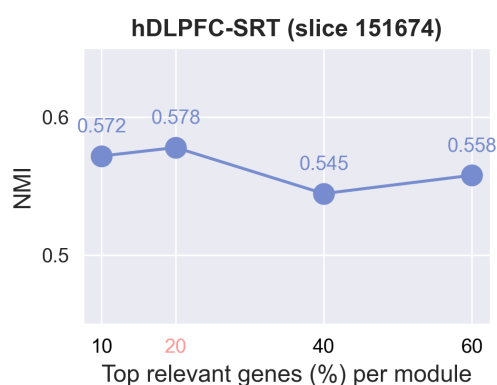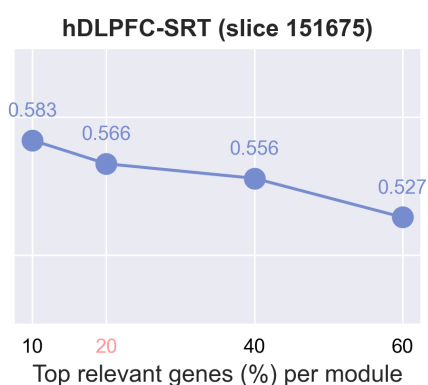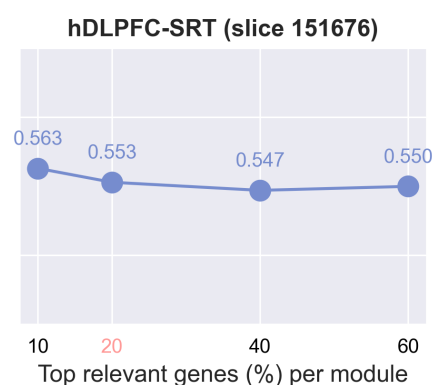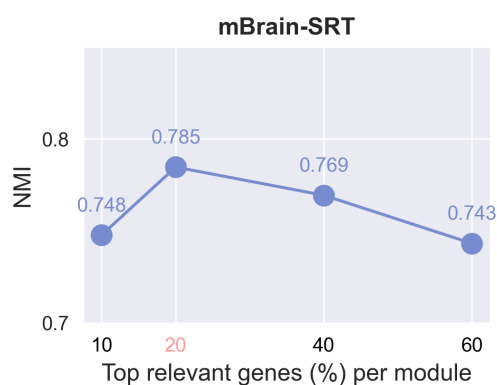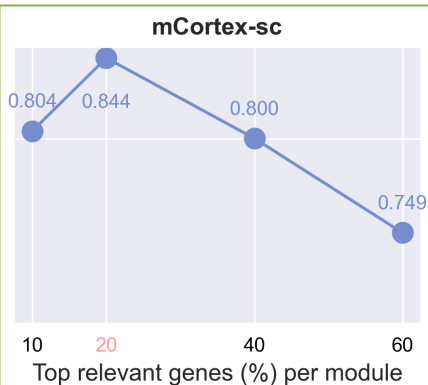

Supplement: qzaf056_Supplementary_Data [file qzaf056_supplementary_data.zip › Figure_S3.pdf]

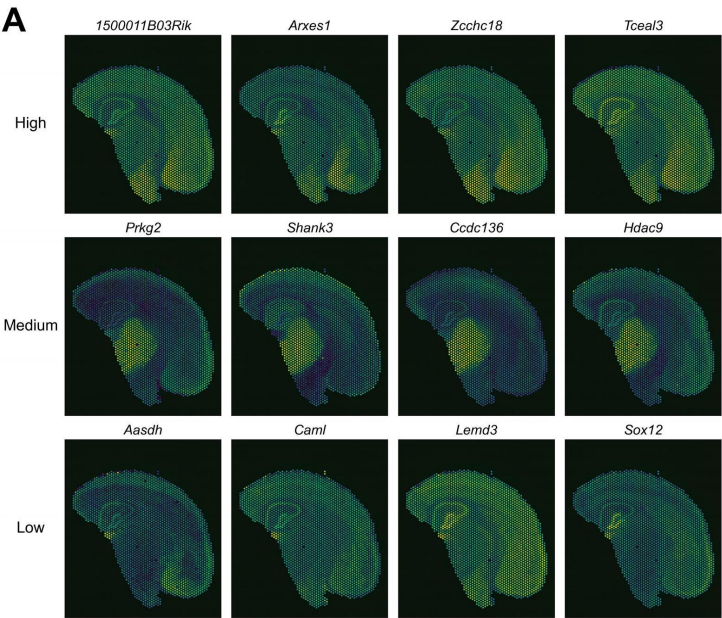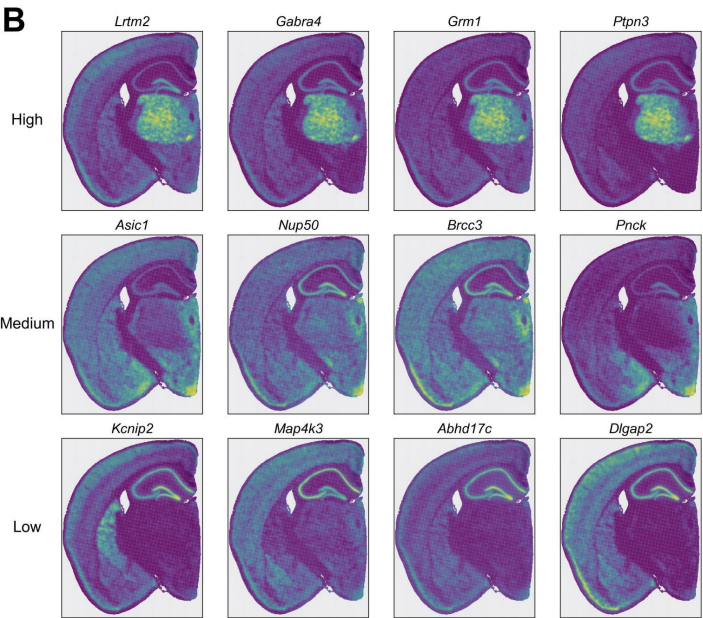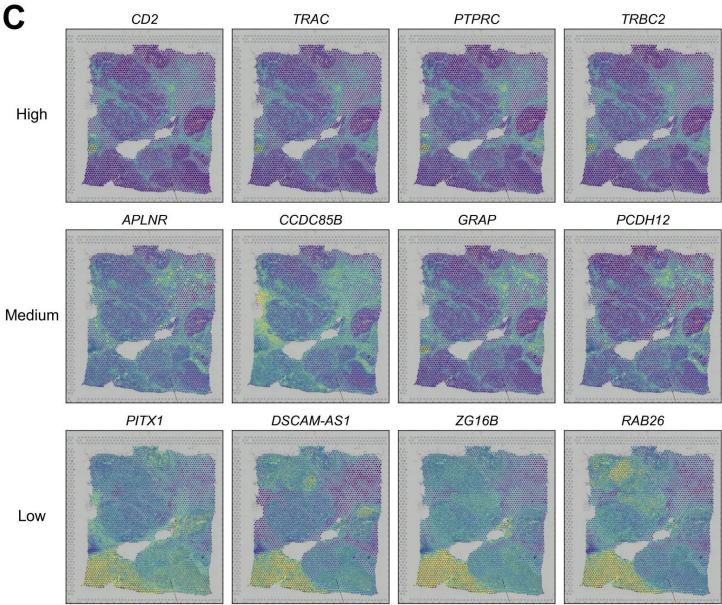

Supplement: qzaf056_Supplementary_Data [file qzaf056_supplementary_data.zip › Figure_S4.pdf]

**A**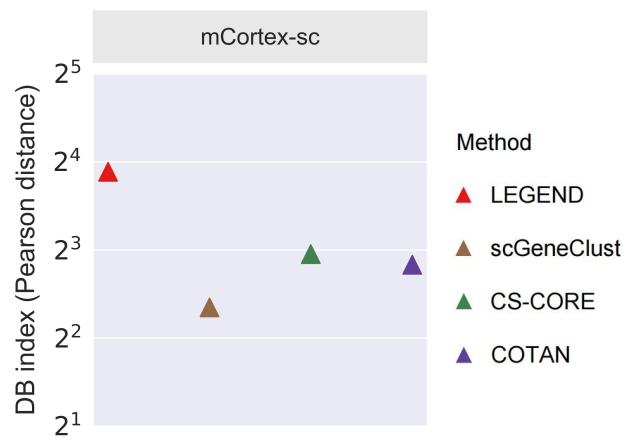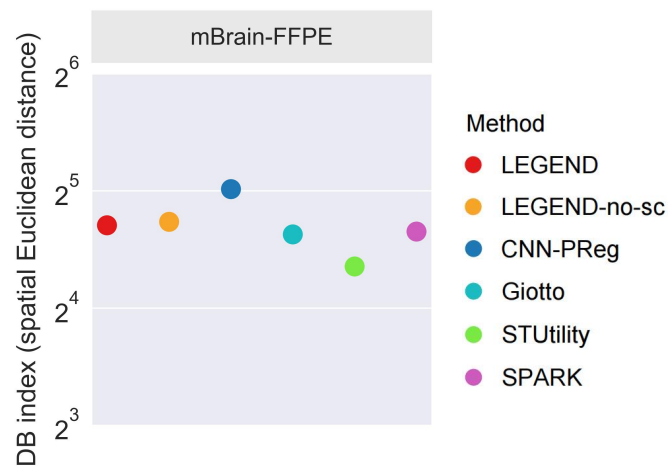**B**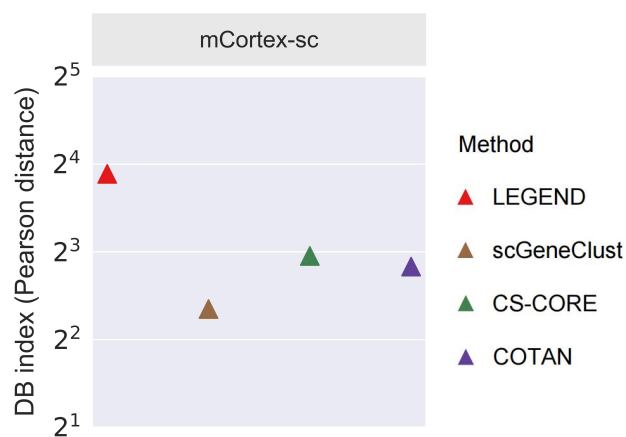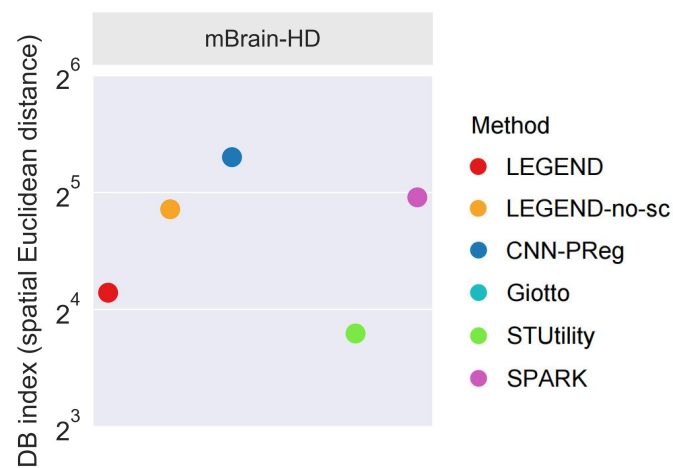**C**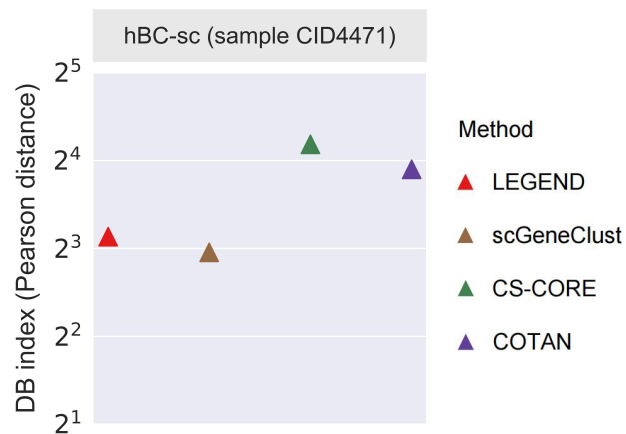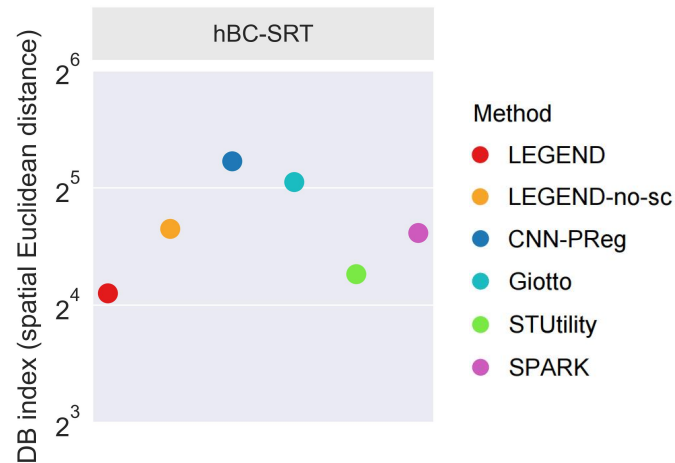

Supplement: qzaf056_Supplementary_Data [file qzaf056_supplementary_data.zip › Figure_S5.pdf]

**A**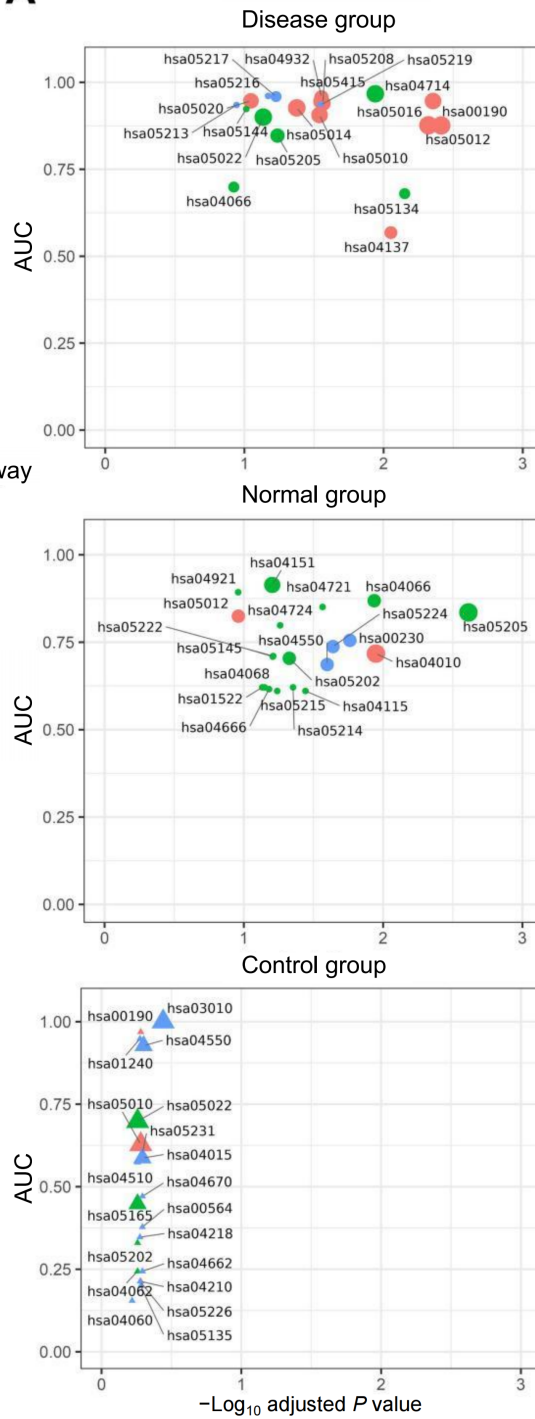**B**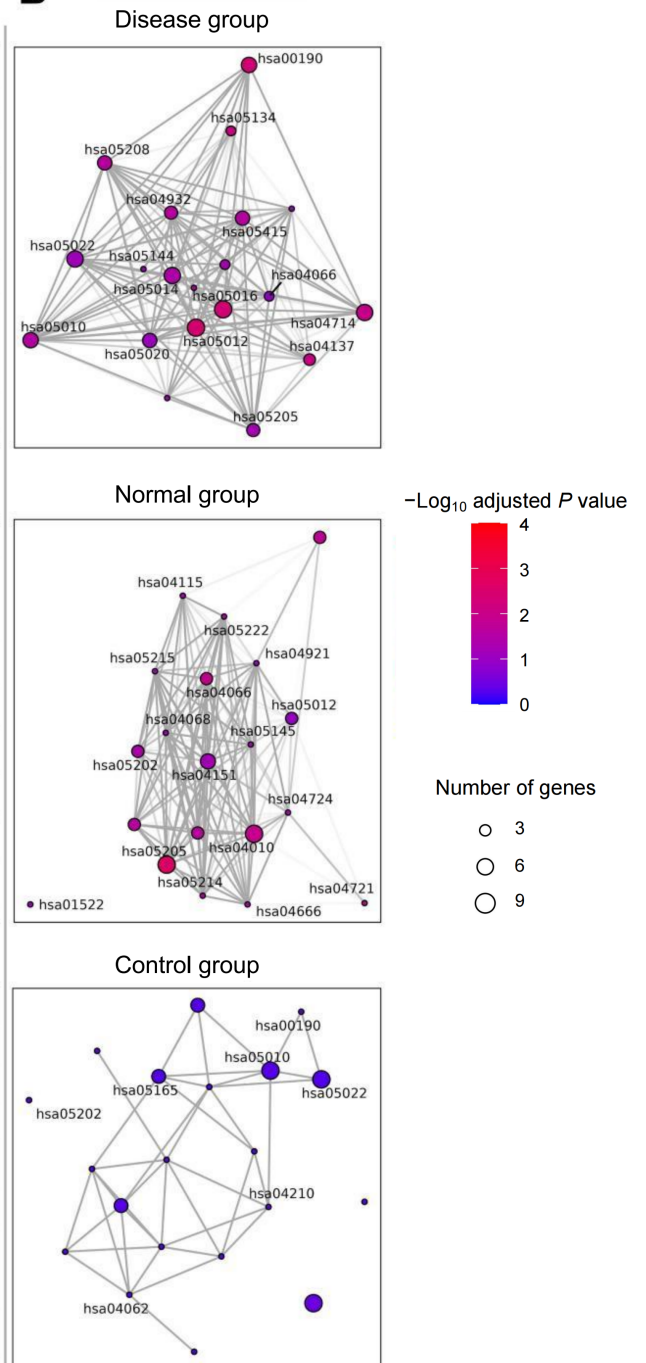

Supplement: qzaf056_Supplementary_Data [file qzaf056_supplementary_data.zip › Figure_S6.pdf]

# A

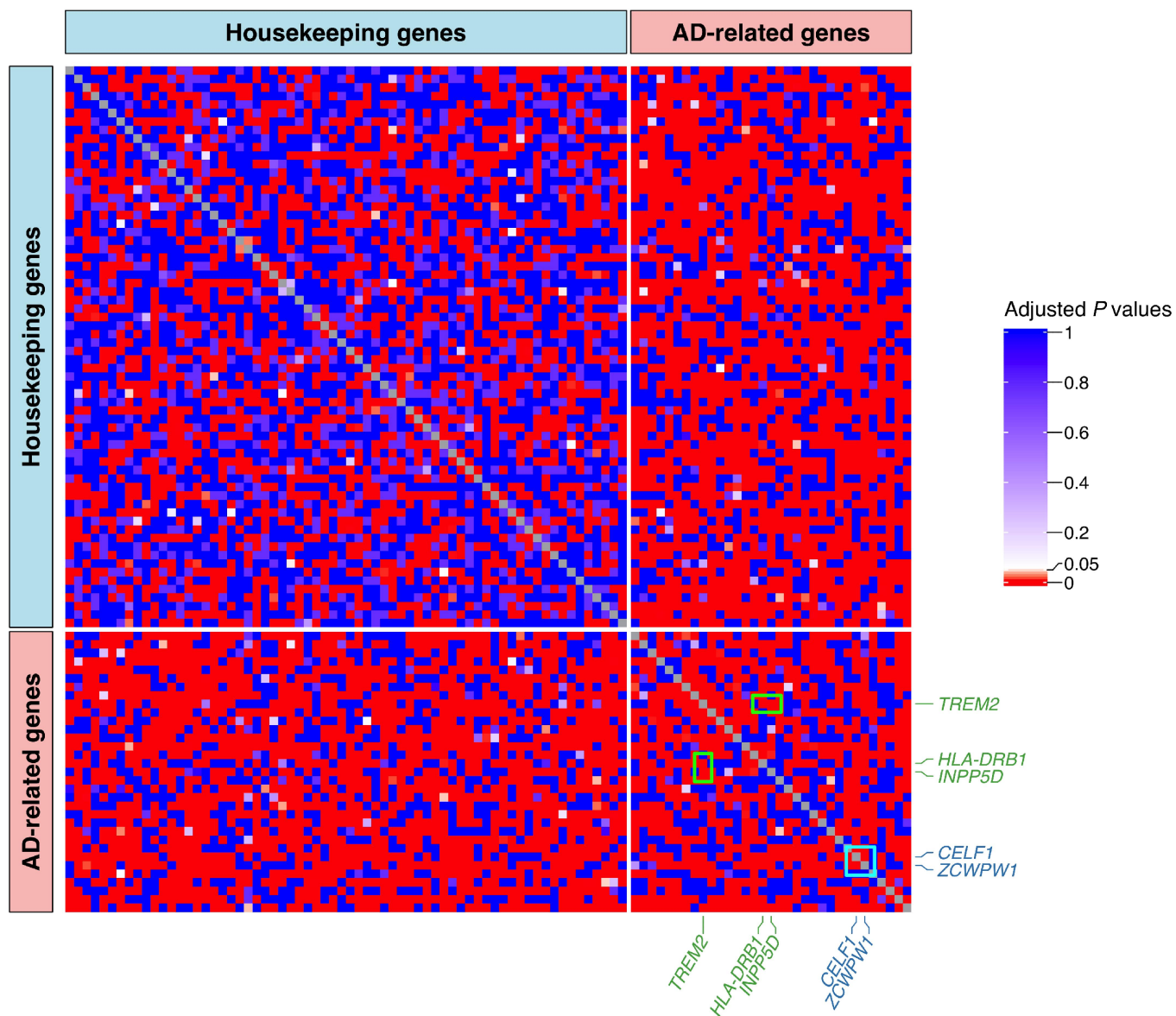

# B

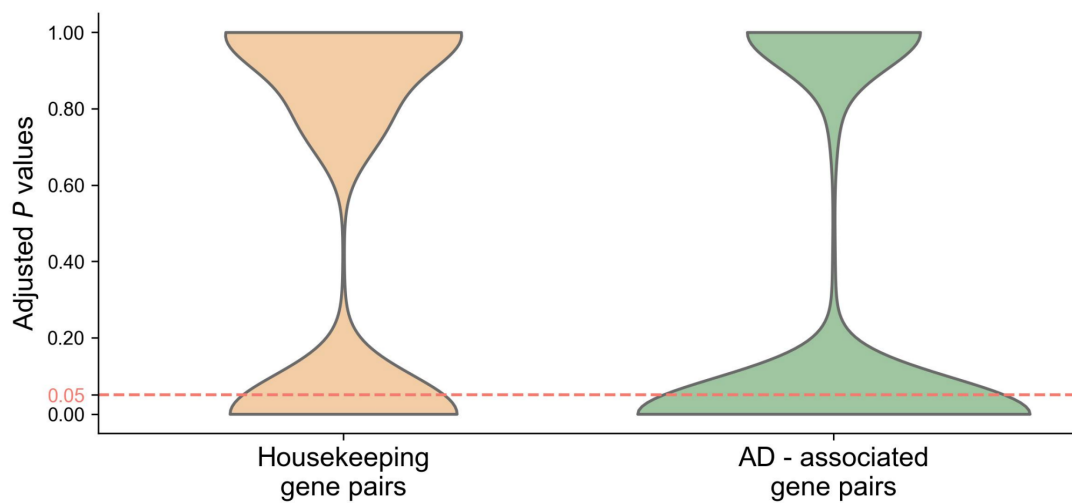

Supplement: qzaf056_Supplementary_Data [file qzaf056_supplementary_data.zip › Figure_S7.pdf]

# SpaGCN (spatial clustering) & Seurat v5 (sing-cell clustering)

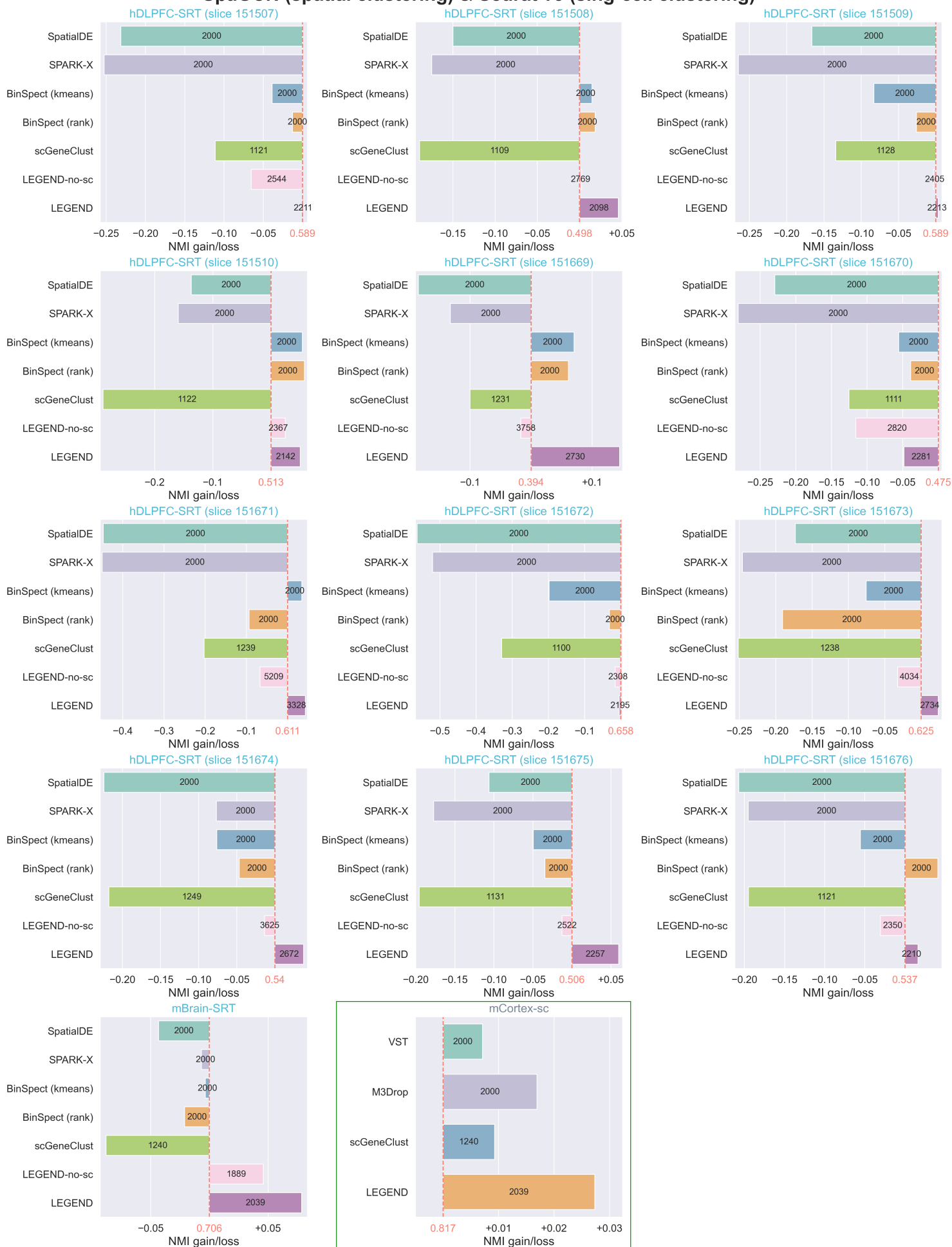

Supplement: qzaf056_Supplementary_Data [file qzaf056_supplementary_data.zip › Figure_S8.pdf]

# Leiden (spatial clustering)

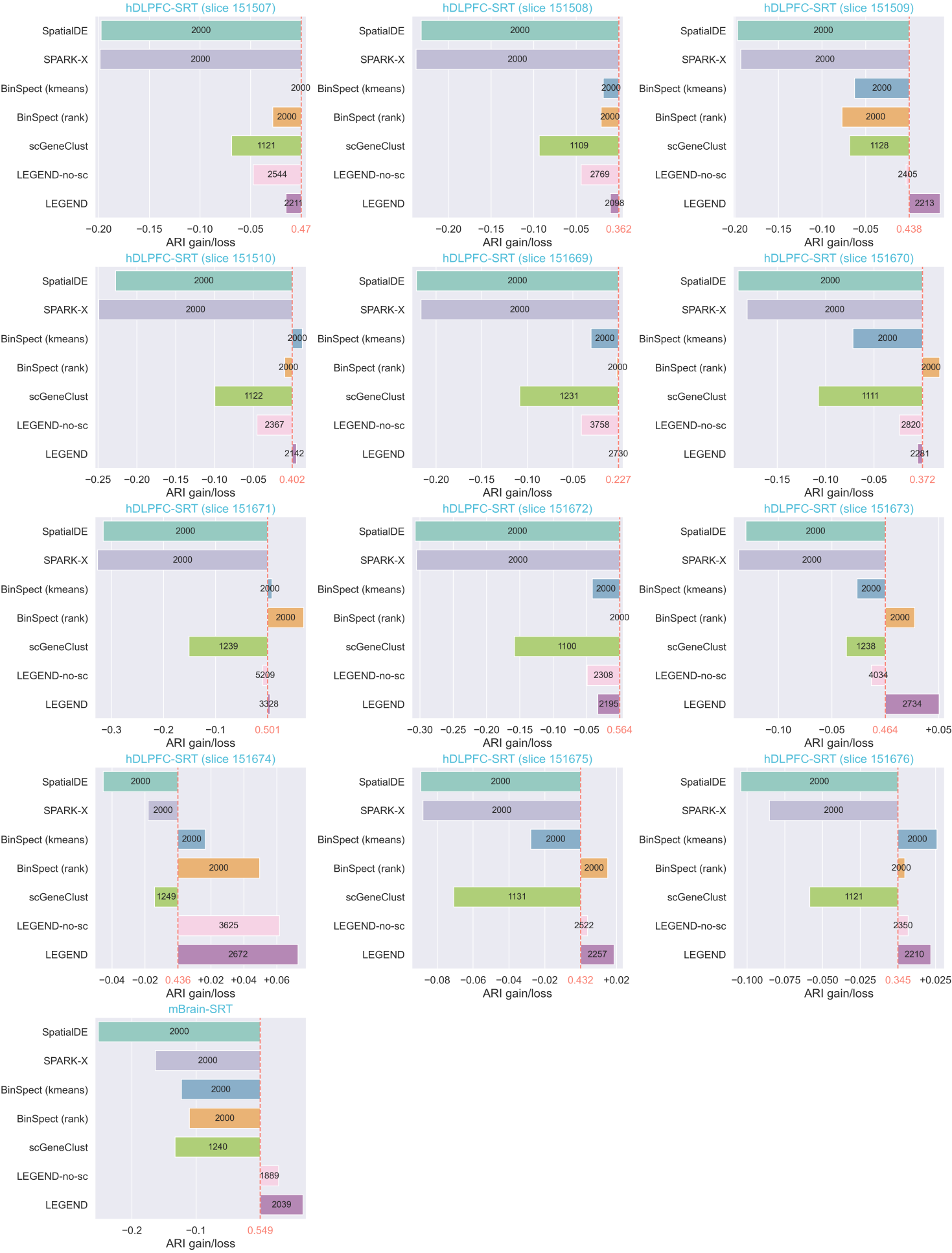

Supplement: qzaf056_Supplementary_Data [file qzaf056_supplementary_data.zip › Figure_S9.pdf]
